# A pan-cancer analysis of the oncogenic role of ERCC6L

**DOI:** 10.1186/s12885-022-10452-3

**Published:** 2022-12-22

**Authors:** Zhimin Lu, Lihong Fei, Guoxin Hou

**Affiliations:** 1 Department of Outpatient, The First Hospital of Jiaxing, Affiliated Hospital of Jiaxing University, Jiaxing, Zhejiang China; 2 Department of Gastroenterology, The First Hospital of Jiaxing, Affiliated Hospital of Jiaxing University, Jiaxing, Zhejiang China; 3 Department of Oncology, The First Hospital of Jiaxing, Affiliated Hospital of Jiaxing University, Jiaxing, Zhejiang China

**Keywords:** ERCC6L, Pan-cancer, Prognosis, Biomarker, Immunity

## Abstract

**Background:**

Excision repair cross-complementation group 6 like (ERCC6L), a polo-like kinase 1 (PLK1)-interacting checkpoint helicase, confers a high risk of cancer and enhances the progression of a variety of cancers. The present investigation aimed to elucidate the pan-cancer expression patterns of ERCC6L and to examine the possibility of using this gene for patient diagnosis and prognosis.

**Methods:**

The expression patterns of ERCC6L in normal and cancer patients at various clinical stages were explored based on TCGA datasets. Subsequently, Bioinformatics techniques were then used to analyze patient’s survival probabilities, Cox multivariate clinical parameters, Gene Ontology (GO) and Kyoto Encyclopedia of Genes and Genomes (KEGG) terms related to ERCC6L, the correlation between mRNA expression levels and patient survival, genetic alterations or somatic mutations of ERCC6L, and immune infiltration.

**Results:**

Most cancer types had higher ERCC6L mRNA levels than normal tissue. Higher ERCC6L expression levels were correlated with poor prognosis for cancer patients. Thus, ERCC6L may serve as an effective diagnostic and prognostic marker for multiple cancers. Moreover, ERCC6L expression levels were higher in patients with higher clinical tumor grades and were associated with poor prognoses at these stages. GO and KEGG analyses revealed a correlation between ERCC6L expression levels and chromatin and cell cycle events. We also found that the mRNA expression level of the ERCC6L promoter and a favorable prognosis was negatively correlated with the promoter’s methylation but not with copy number variation. A quantitative analysis of immune infiltration suggested a positive correlation between ERCC6L levels and the infiltration of Th2 immune cells in main cancer types. Finally, we examined the ERCC6L somatic mutations, especially single-nucleotide variants, and ERCC6L expression-related drug sensitivity.

**Conclusions:**

Herein, we reported a comprehensive investigation of the tumor-promoting role of ERCC6L in various cancer types. ERCC6L is a candidate biomarker for diagnosing and unfavorable prognosis of specific cancers.

**Supplementary Information:**

The online version contains supplementary material available at 10.1186/s12885-022-10452-3.

## Introduction

Excision repair cross-complementation group 6-like (ERCC6L), also known as polo-like kinase 1-interacting checkpoint helicase, is a recently discovered DNA helicase belonging to the SNF2 family. Members of this protein family have been demonstrated to participate in the reassembly of ribosomes, RNA processing, and translation initiation [1, 2]. ERCC6L is recruited to catenate centromere-related DNA as a protein that interacts with Plk1 [3]. ERCC6L expression levels are higher in tumors (breast, colorectal, and hepatocellular cancer) than in comparable normal tissue [4], indicating that it can be used as a biomarker for cancer patients. Moreover, ERCC6L has been depicted to exhibit oncogenic properties in colorectal [5], hepatocellular [6], and breast [4] cancer cells, suggesting that it may be a viable target for cancer therapy. Considering that the pan-cancer expression pattern and diagnostic or prognostic value of ERCC6L are yet to be examined, we conducted a series of bioinformatic analyses to analyze the differential expression of ERCC6L during cancer progression, and we investigated whether ERCC6L can be further explored as a biomarker in specific cancer types.

We found that ERCC6L expression was upregulated in patients from various cancer types than in normal tissue samples. We also verified that ERCC6L was associated with poor prognosis in cancer patients and that it may be utilized as an independent prognostic biomarker in certain cancer types. We also systematically studied the association between genetic variants of ERCC6L and immune infiltration in various cancers.

We comprehensively investigated the diagnostic and prognostic roles of ERCC6L in a panel of common cancers. These findings may shed light on other clinical and biological functions of ERCC6L in specific cancer types.

## Materials and methods

### Gene expression analysis of ERCC6L

ERCC6L mRNA expression data were downloaded from The Cancer Genome Atlas (TCGA) database (https://www.cancer.gov/about-nci/organization/ccg/research/structural-genomics/tcga). Differential expression levels of ERCC6L between normal and tumor samples and diverse clinical stages were analyzed. All sequencing and differential gene expression data were normalized using the R edgeR package (V 3.30.3). The threshold was |log2fold-change) |> 1 and false discovery rate < 0.05. Expression data were transformed by calculating log2 (transcripts per million + 1), and plots were generated using the R package. We selected cancer patients in TCGA with clinical information and removed duplicates.

### Survival prognosis based on ERCC6L levels

The survival data and clinical characteristics of patients with specific cancers were downloaded from the TCGA database. Subsequently, Kaplan–Meier plots were generated using the Kaplan–Meier curve R survival package (V3.1.12). A 50% expression level of ERCC6L was set as a threshold to stratify patients as ERCC6L-low or ERCC6L-high.

### Cox regression analysis of overall survival

Multivariate Cox analyses of various clinical factors and ERCC6L expression levels were performed using patient data derived from TCGA. Plots were generated using the R package.

### Receiver operating characteristic (ROC) curve analysis of cancer-related survival

ROC curve analysis was performed using the pROC package in R to evaluate the diagnostic performance of ERCC6L. Patients were categorized into low and high expression categories, and ROC curves were constructed based on patient survival data.

### Genes co-expressed with ERCC6L

We used TCGA data for nine different cancer types and examined the co-expressed genes (correlations < -0.5 or >  + 0.5) to create an ERCC6L co-expression network. After overlapping the identified genes in all the cancers examined, 197 genes were found that co-expressed with ERCC6L. The R package, clusterProfiler, was then used to perform Gene Ontology (GO) and Kyoto Encyclopedia of Genes and Genomes (KEGG) [7–9] analyses of cancer-related biological processes or pathways affected by ERCC6L.

### Protein–protein interaction network analysis

The input of ERCC6L and 197 co-expressed genes into STRING (https://cn.string-db.org/) yielded 195 genes having interaction. C5orf34, AC099850.3, and AC091057.1, which did not interact with them, were eliminated. The key subnetworks were filtered out using the molecular complex detection (MCODE) plugin in Cytoscape (V3.9.1) software using the default parameters. Cluster1 had 131 genes (ERCC6L is in this largest sub-network). Hub gene (top 10) was identified from the 131 genes using the Cytohubba plug-in and MCC algorithm.

### Genetic variants and somatic mutation analysis of ERCC6L

Promoter methylation data were derived from the DNA Methylation Interactive Visualization Database [10]. Copy number variation (CNV) and other genomic variant data were derived from the cBioportal and Gene Set Cancer Analysis (GSCA) databases (http://bioinfo.life.hust.edu.cn/GSCA/#/) [11, 12], and their correlations with ERCC6L mRNA expression levels and patient survival were analyzed.

### Immune infiltration score analysis

Immune-infiltration estimates were performed for protein-coding type genes in 9 different types of tumor samples, and the input data type was TPM. The Pearson correlation coefficients were calculated between the three scores, tumor purity and ERCC6L log2 (TPM + 1). Correlation line bar graphs were drawn using the ggplot2 (V 3.3.6) in the R package (V4.0.5).

### Immune cell infiltration analysis

We determined the tumor purity of each patient using the R packages GSVA and GSEABase, based on the single sample gene set enrichment analysis (ssGSEA) algorithm, to quantify the infiltration of a diverse range of immune cells [13]. The R package estimate was used to determine the correlations between gene expression levels and immune cell infiltration based on the ESTIMATE algorithm. The pheatmap package was used to create a heatmap. Partial correlation values and *p-*values were calculated using Spearman’s rank correlation analysis.

### The correlation between ERCC6L and tumor mutation burden, microsatellite instability, and neoantigens

SNV and CNV data of primary tumor and metastatic samples were downloaded using TCGAbiolinks (V2.25.0) in the R package (V4.0.5). The tumor mutational burden (TMB) of each tumor sample was calculated using the mafTools (V2.4.12) package. Clinical data were downloaded from the cBioPortal website (https://www.cbioportal.org/), which included the microsatellite instability (MSI) score of each tumor sample. Neoantigen count data were downloaded from https://gdc.cancer.gov/about-data/publications/panimmune. TMB, MSI, and neoantigen Pearson correlation coefficients were calculated with ERCC6L LOG2 (TPM + 1), respectively. A rose plot was drawn using the ggplot2 (V 3.3.6) package in the R package (V4.0.5).

### Statistical analysis

Correlations between variables were explored using Pearson or Spearman coefficients. Continuous variables fitting a normal distribution between binary groups were compared using a t-test; otherwise, the Mann–Whitney U test was applied. Categorical variables were compared using the chi-square test or Fisher’s exact test. Survival curves for prognostic analyses of categorical variables were generated using the Kaplan–Meier method, while the log-rank test was applied to estimate statistical significance. The significance level was set at *P* < 0.05, and all statistical tests were two-sided. All statistical data analyses were implemented using R software (V3.6.3).

## Results

### ERCC6L gene expression analysis

**Fig. 1:**
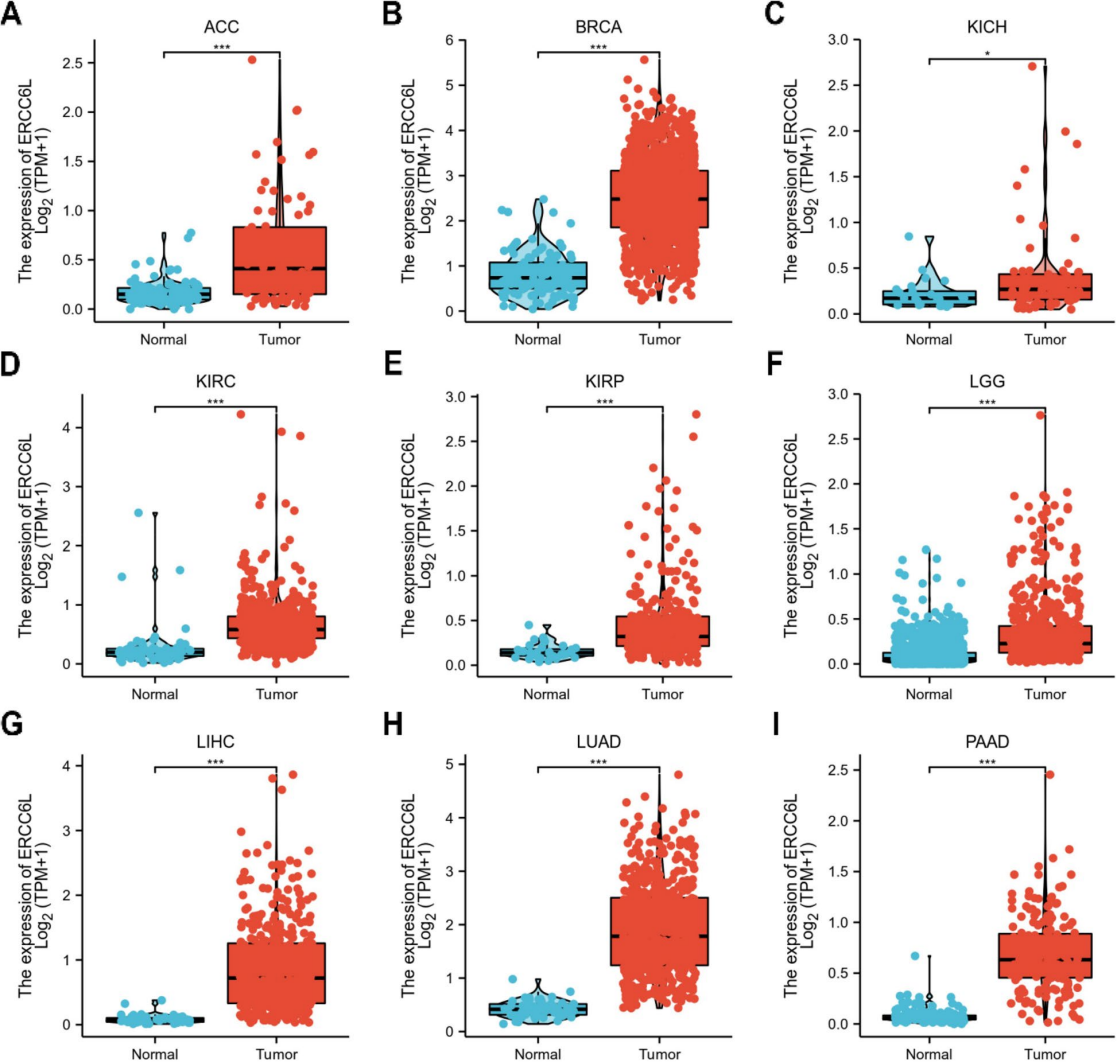
ERCC6L expression in diverse cancer types. **A-I** Differential expression analysis of ERCC6L in multiple cancers, including adrenocortical carcinoma (ACC, **A**), invasive breast carcinoma (BRCA, **B**), chromophobe renal cell carcinoma (KICH, **C**), clear-cell renal cell carcinoma (KIRC, **D**), papillary renal cell carcinoma (KIRP, **E**), lower grade glioma (LGG, **F**), hepatocellular carcinoma (LIHC, **G**), lung adenocarcinoma (LUAD, **H**), and pancreatic adenocarcinoma (PAAD, **I**). Statistical analyses were performed to compare ERCC6L levels in normal and tumor samples

We began our study by examining the mRNA expression levels of ERCC6L in commonly diagnosed cancers. Compared with its expression levels in normal tissue compartments, ERCC6L was expressed at significantly higher levels in tumor samples from patients with cancers, including adrenocortical carcinoma (ACC), invasive breast carcinoma (BRCA), kidney chromophobe (KICH), kidney renal clear cell carcinoma (KIRC), kidney renal papillary cell carcinoma (KIRP), brain lower grade glioma (LGG), liver hepatocellular carcinoma (LIHC), lung adenocarcinoma (LUAD), pancreatic adenocarcinoma (PAAD), bladder urothelial carcinoma (BLCA), cervical squamous cell carcinoma and endocervical adenocarcinoma (CESC), colon adenocarcinoma (COAD), cholangiocarcinoma (CHOL), lymphoid neoplasm diffuse large B-cell lymphoma (DLBC), esophageal carcinoma (ESCA), glioblastoma multiforme (GBM), head and neck squamous cell carcinoma (HNSC), ovarian cancer (OV), prostate adenocarcinoma (PRAD), testicular germ cell tumors (TGCT), thyroid carcinoma (THCA), uterine carcinosarcoma (UCS), lung squamous cell carcinoma (LUSC), rectum adenocarcinoma (READ), stomach adenocarcinoma (STAD), thymoma (THYM), skin cutaneous melanoma (SKCM) and uterine corpus endometrial carcinoma (UCEC) (Figs. 1A–I, S1A–H, J–N, and S2A–F). Notably, we also found that ERCC6L levels were specifically upregulated in tumor samples than paired normal tissue samples from the same individual in patients with LUSC, READ, STAD, UCEC, BRCA, KICH, KIRC, KIRP, LIHC, and LUAD (Figs. S2G–J and S3A–F). However, decreased ERCC6L levels were observed in patients with acute myeloid leukemia (LAML) (Fig. S1I). Additionally, we examined the ERCC6L protein levels in multiple common cancers. ERCC6L protein was elevated in eight of nine cancers that we investigated (Fig. S3). These results suggested that the expression of ERCC6L is elevated at both mRNA and protein levels in most of the cancers examined.

### Prognostic value of ERCC6L for patient survival

**Fig. 2:**
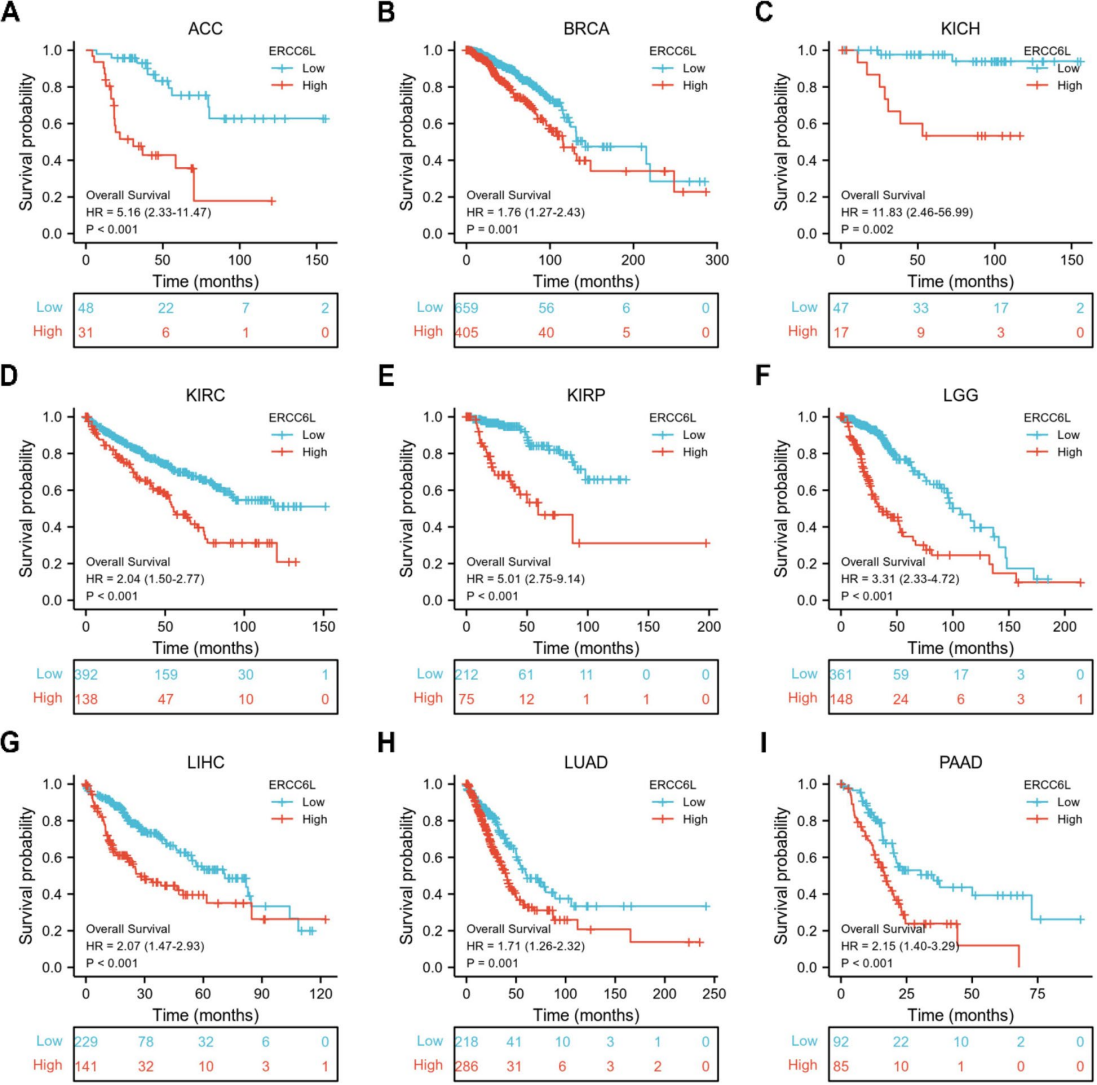
High ERCC6L levels were correlated with a poor prognosis for cancer patients. **A-I** Overall survival analysis of cancer patients stratified by ERCC6L expression level. The patients were diagnosed with ACC (**A**), BRCA (**B**), KICH (**C**), KIRC (**D**), KIRP (**E**), LGG (**F**), LIHC (**G**), LUAD (**H**), or PAAD (**I**)

**Fig. 3:**
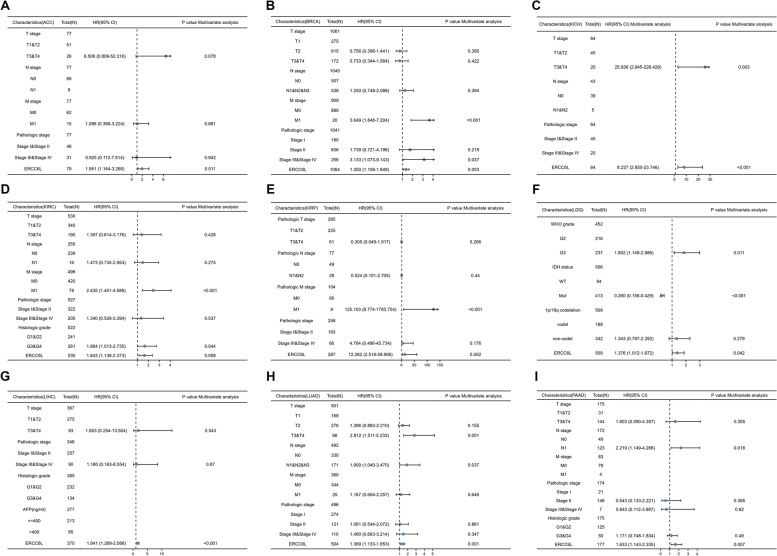
Multivariate Cox regression hazards analysis of the overall survival of cancer patients. The results were categorized according to cancer types, ACC (**A**), BRCA (**B**), KICH (**C**), KIRC (**D**), KIRP (**E**), LGG (**F**), LIHC (**G**), LUAD (**H**), and PAAD (**I**)

**Fig. 4:**
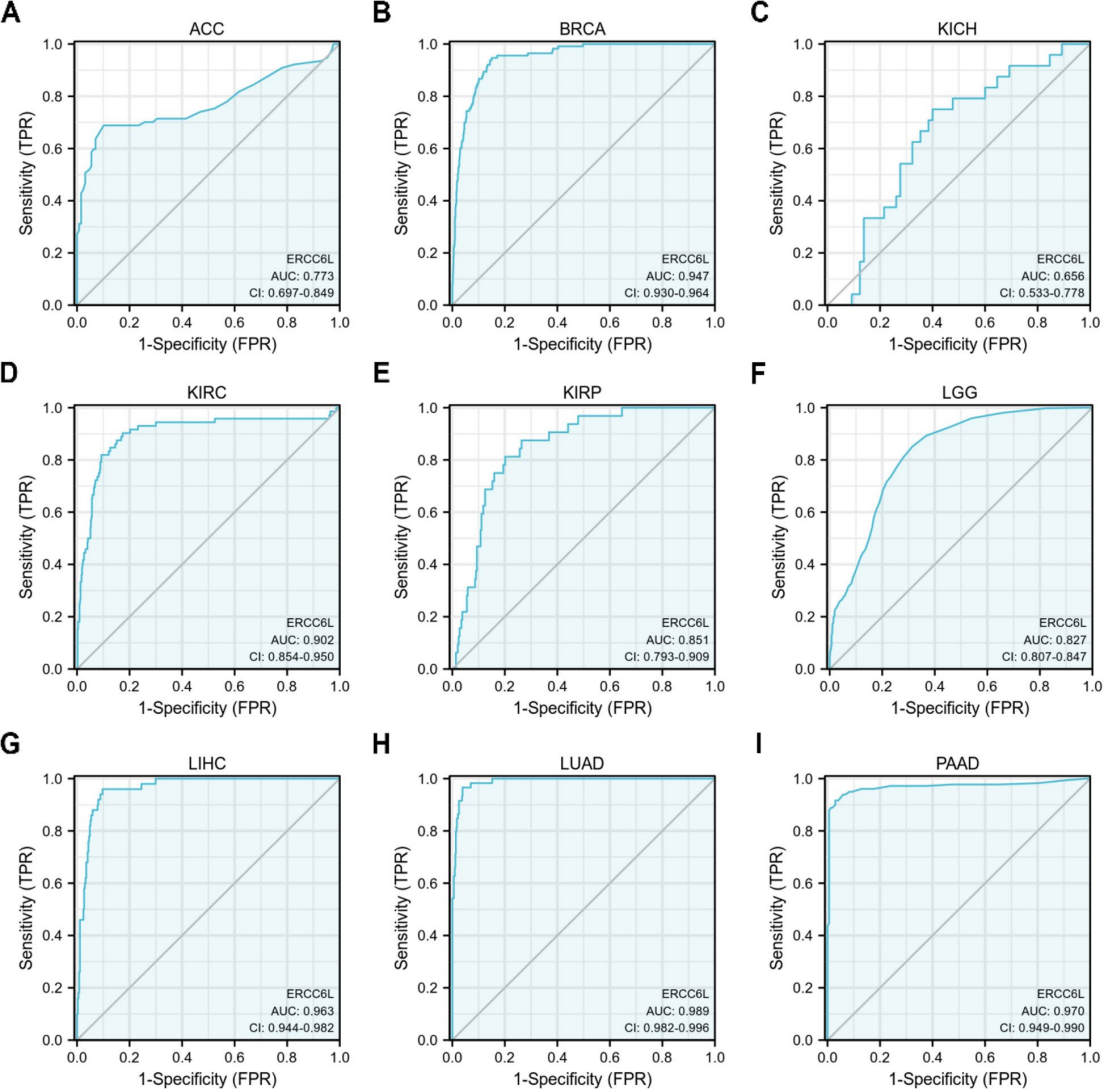
Estimated ROC curve for determining the diagnostic value (based on sensitivity and specificity) of ERCC6L in multiple cancer types. The results were categorized according to cancer types, ACC (**A**), BRCA (**B**), CRCC (**C**), ccRCC (**D**), PRCC (**E**), LGG (**F**), HCC (**G**), LUAD (**H**), and PAAD (**I**)

We investigated whether ERCC6L could be used as a biomarker for the prognosis of cancer patients, given the pattern of ERCC6L upregulation in most cancers. As expected, high ERCC6L levels significantly correlated with unfavorable outcomes in patients with ACC, BRCA, KICH, KIRC, KIRP, LGG, LIHC, LUAD, PAAD, UCEC, SKCM, MESO, Sarcoma (SARC), and Uveal Melanoma (UVM) (Figs. 2A–I and S4A–F). However, the ERCC6L expression level was not correlated with the overall survival (OS) of patients with BLCA, CESC, CHOL, COAD, DLBC, ESCA, GBM, HNSC, LAML, OV, Pheochromocytoma/paraganglioma (PCPG), PRAD, TGCT, THCA, or UCS (Figs. S5A–O). Moreover, ERCC6L levels were negatively associated with higher survival rates in some cancer patients, such as LUSC, READ, STAD, and THYM (Figs. S6A–D). As clinical stages, like the T and N stages, are valuable parameters for predicting a patient’s survival, we sought to determine whether the ERCC6L expression level could also function as an independent predictor of survival. Surprisingly, the ERCC6L expression level was found to be a potential predictor of survival in patients with ACC, BRCA, KICH, KIRC, KIRP, LGG, LIHC, LUAD, and PAAD in multivariate and univariate Cox analyses of various clinical factors or its expression levels (Figs. 3A–I and S7A–I). Furthermore, the estimated ROC curve illustrated that the diagnostic sensitivity and specificity of ERCC6L expression levels were effective in patients with ACC, BRCA, KICH, KIRC, KIRP, LGG, LIHC, LUAD and PAAD (area under the curve > 0.6; Figs. 4A–I). Importantly, we observed that, in addition to OS, high levels of ERCC6L expression were associated with lower disease-specific survival and progression-free intervals in nine cancer types (i.e., ACC, BRCA, KICH, KIRC, KIRP, LGG, LIHC, LUAD, and PAAD; Figs. S8A–I and S9A–I).

### Expression and prognostic value of ERCC6L for cancer patient survival in specific clinical stages

**Fig. 5:**
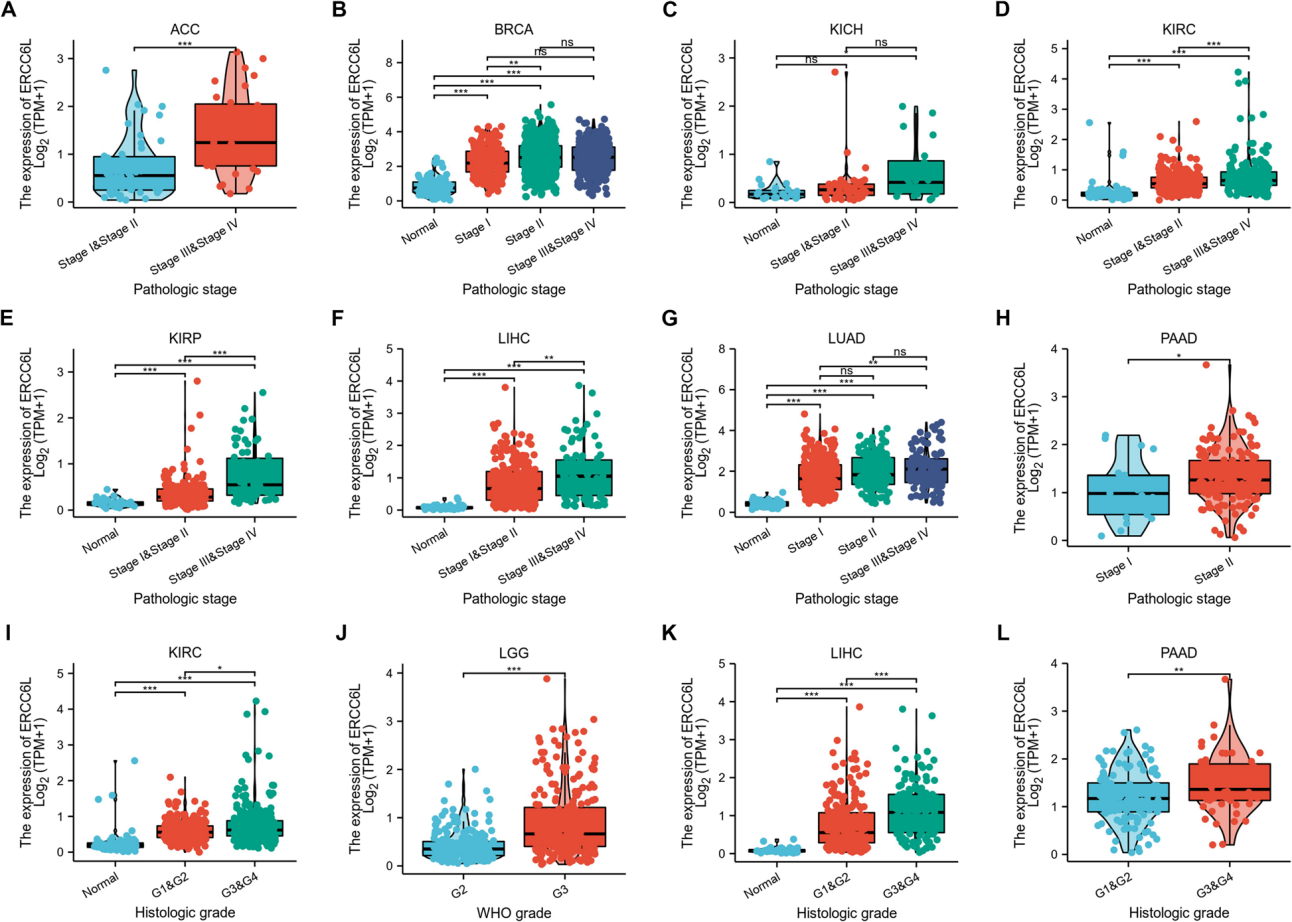
ERCC6L expression levels in various clinical cancer stages and grades. **A-F** Differential expression analysis of ERCC6L in diverse clinical stages and grades (pathological stages, histological grades, or World Health Organization stages) of indicated cancers

**Fig. 6:**
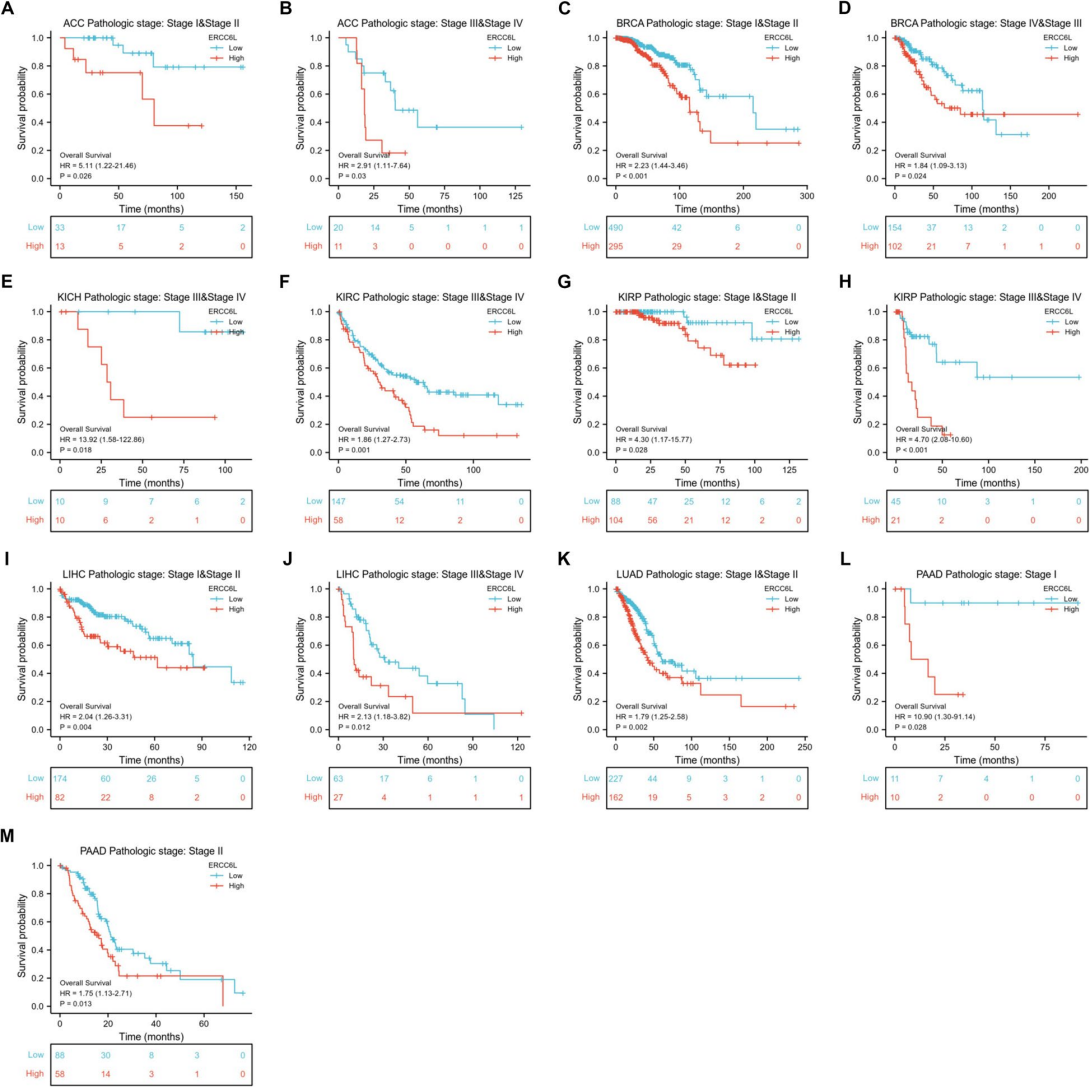
ERCC6L was associated with unfavorable outcomes in cancer patients at different clinical stages. Overall survival of cancer patients stratified by ERCC6L expression levels. The patients were classified according to indicated clinical stages of different cancers

Then, we determined whether ERCC6L was also aberrantly expressed in various clinical stages of cancer and correlated with the survival of patients when classified into certain stages. Interestingly, we found that ERCC6L was generally more expressed in higher pathologic stages/grades (stage III or IV vs. stage I or II and grade 3, grade 4 vs. grade 1 or grade 2, T3 or T4 vs. T1 or T2, N2 or N3 vs. N1 or N0, and M1 vs. M0) in patients with the nine cancer types mentioned above (Figs. 5A–L, S10A–H, S11A–H, and S12A–H). In addition to the entire patient cohort, ERCC6L was associated with poor outcomes in the nine types of cancer patients at certain stages (Figs. 6A–M, S13A–E, S14A–L, S15A–J, and S16A–E). These findings imply that ERCC6L is upregulated in late pathological stages/status and is related to low OS rates when cancer patients are classified into subgroups based on their clinical parameters.

### Analysis of ERCC6L-regulated processes

**Fig. 7:**
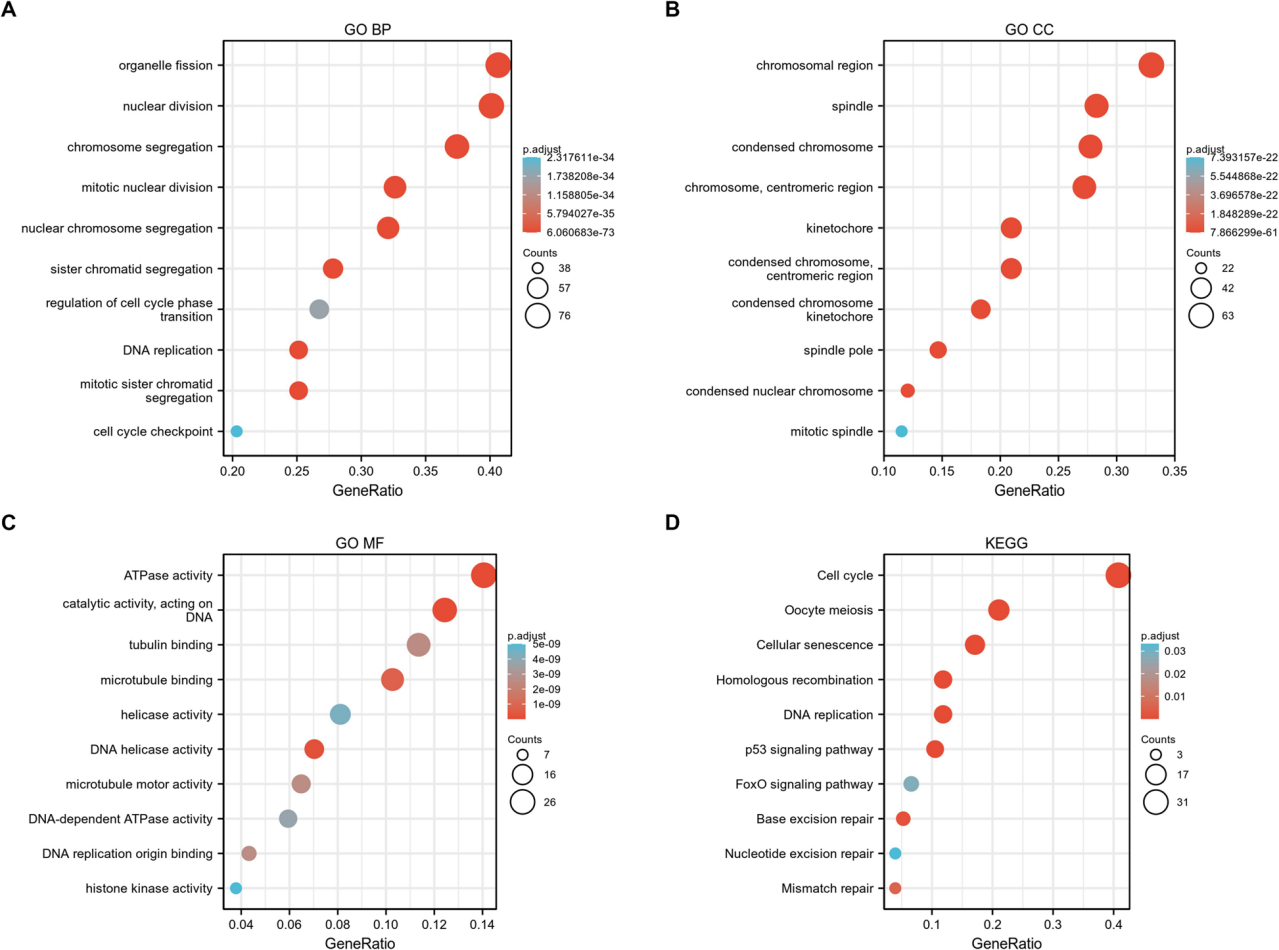
Gene ontology and Kyoto encyclopedia of genes and genomes analyses of genes co-expressed with ERCC6L. **A–C** Gene ontology analysis of biological processes (BP; **A**), cellular components (CC; **B**), and molecular functions (MF; **C**) correlated with ERCC6L expression levels. **D** KEGG analysis of biological pathways correlated with ERCC6L expression levels

**Fig. 8:**
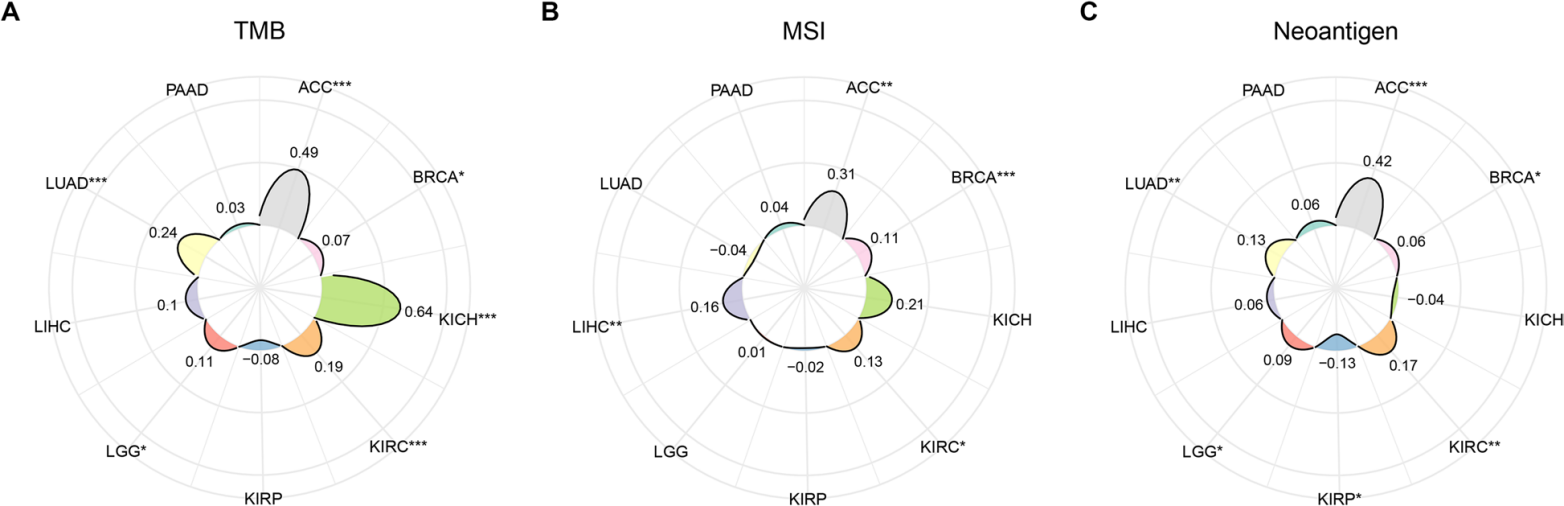
Correlations between ERCC6L and tumor mutation burden, microsatellite instability, and neoantigens. Associations between ERCC6L and tumor mutation burden (TMB, **A**), microsatellite instability (MSI, **B**), and neoantigens (**C**)

Next, we determined the biological processes or signaling pathways associated with the expression level of ERCC6L in cancers. To this end, overlapping co-expressed genes in the nine cancer types were used to identify a panel of ERCC6L-correlated genes (a total of 197) (Figs. S17A and B). First, the hub gene analysis among the 197 genes and protein–protein interaction network analysis was carried out to unravel the physiological function of ERCC6L more accurately (Figs. S18A and B). Subsequently, GO analysis was performed based on this set of ERCC6L-correlated genes. The three most highly correlated biological processes include organelle fission, nuclear division, and chromosome segregation (Fig. 7A). This gene set was also enriched in the cellular components, chromosome region, spindle, and condensed chromosomes (Fig. 7B). Moreover, the most correlated molecular functions of ERCC6L were found to be ATPase activity and catalytic activity, acting on DNA and tubulin binding (Fig. 7C). Furthermore, KEGG analysis demonstrated that the cell cycle was the key process regulated by ERCC6L in cancer patients (Fig. 7D). Importantly, we have analyzed the correlations between ERCC6L and tumor mutation burden (TMB), microsatellite instability (MSI) and neoantigens in these nine cancers (Fig. 8). We did find statistically significant associations in some cancer types. We systematically studied the processes and pathways correlated with ERCC6L expression in nine cancers.

### Analysis of ERCC6L genetic variants

**Fig. 9:**
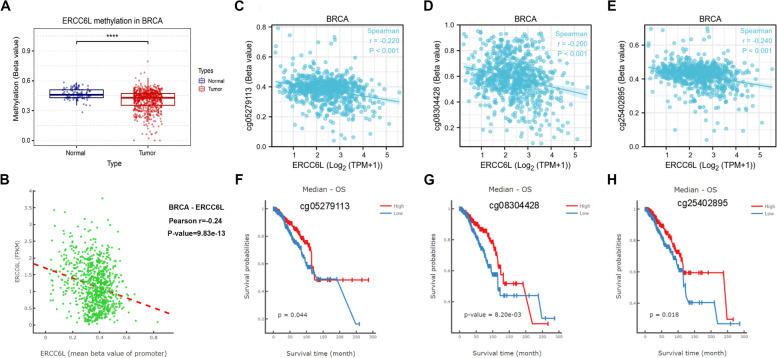
Methylation of ERCC6L promoter was correlated with improved prognosis in BRCA patients. **A** Comparison of ERCC6L promoter methylation levels in normal tissue and BRCA tumor samples. **B** Correlation analysis of ERCC6L promoter methylation and mRNA expression levels in BRCA cohorts. **C**, **D** Correlation analysis of the indicated ERCC6L promoter methylation sites and ERCC6L mRNA expression levels in BRCA cohorts. **F–H** Overall survival analysis of BRCA patients stratified by ERCC6L promoter methylation levels at three methylation sites

We then investigated ERCC6L genetic alterations in cancer patients. First, we analyzed CNV in the nine cancer types mentioned above. Most of the tested cancer types did not significantly differ in ERCC6L expression levels with different groups of CNV; similarly, ERCC6L mRNA expression levels did not significantly correlate with ERCC6L CNVs (Figs. S17A–I and S18A–I). Unexpectedly, we observed that in KIRP, CNV was negatively associated with ERCC6L mRNA expression levels and patients with a loss of CNV (indicating a decrease in ERCC6L mRNA levels) had poor clinical outcomes (OS, disease-free interval [DFS], progression-free survival [PFS], and disease-specific survival [DSS]; Figs. S18E and S19A–D). Subsequently, we determined if the changes in ERCC6L mRNA levels were related to the methylation of its promoter. Most cancer types, excluding PAAD, exhibited a negative correlation between ERCC6L mRNA expression levels and promoter methylation (Figs. S20A–I). In particular, a significant inverse correlation was observed for BRCA (Fig. S20B). However, we also observed a significant reduction in ERCC6L promoter methylation levels in BRCA tumors than in normal control tissue (Fig. 9A) and a negative correlation between ERCC6L mRNA levels and methylation status in BRCA patients (Fig. 9B). Furthermore, we found an inverse association between ERCC6L expression levels and methylation at the three most prevalent methylation sites in the promoter region, cg05279113, cg08304428, and cg25402895 (Figs. 9C–E). Notably, unlike ERCC6L mRNA levels, its hypermethylation was associated with a favorable prognosis (OS) for BRCA patients (Figs. 9F–H). Our results demonstrated that promoter hypomethylation may significantly cause elevated ERCC6L levels in BRCA patients and was associated with lower patient survival rates.

### Immune cell infiltration analysis

**Fig. 10:**
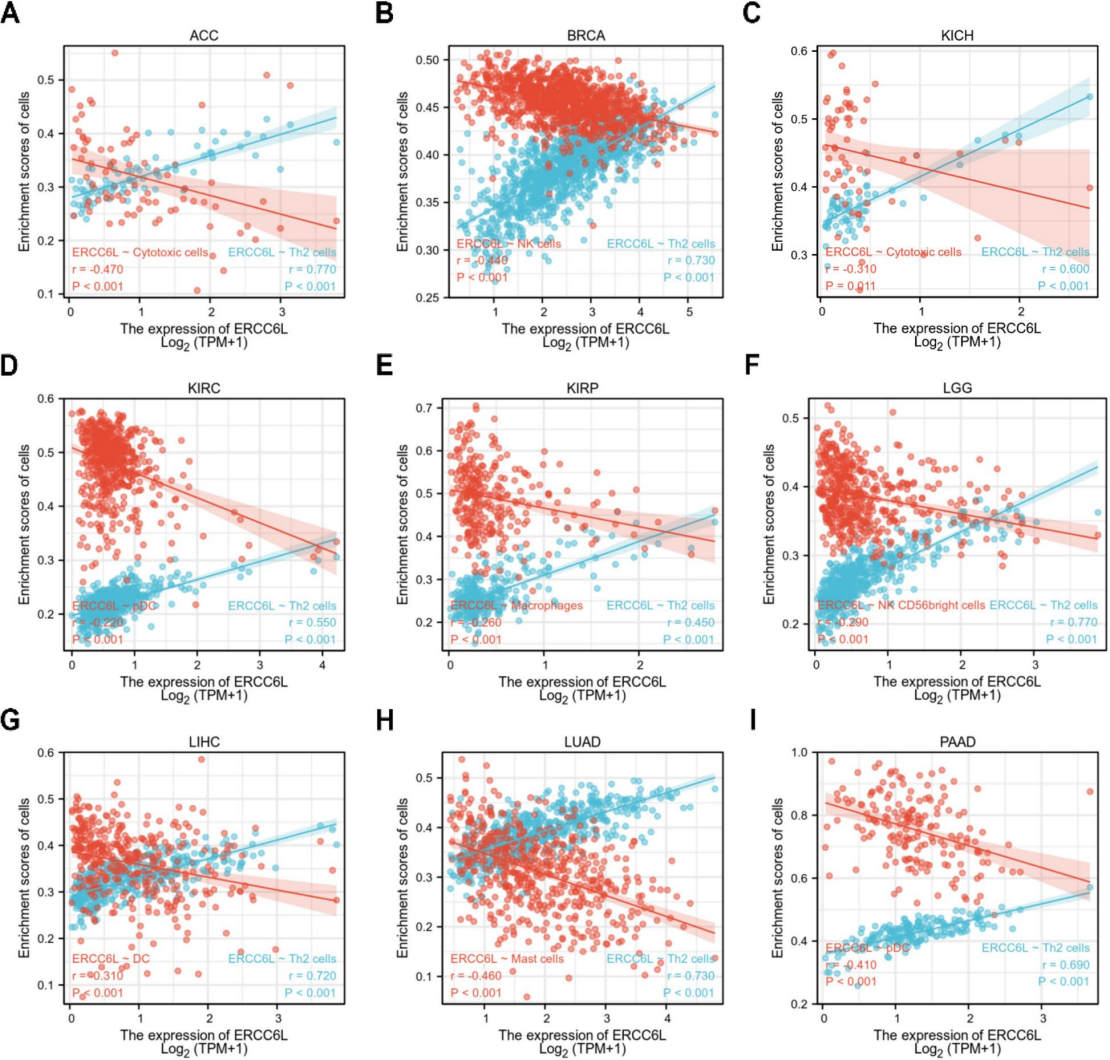
The correlation between ERCC6L expression levels and immune cell infiltration in diverse cancer types. **A–I** The correlation between ERCC6L expression levels and infiltration of the indicated immune cells in the indicated cancer types

**Fig. 11:**
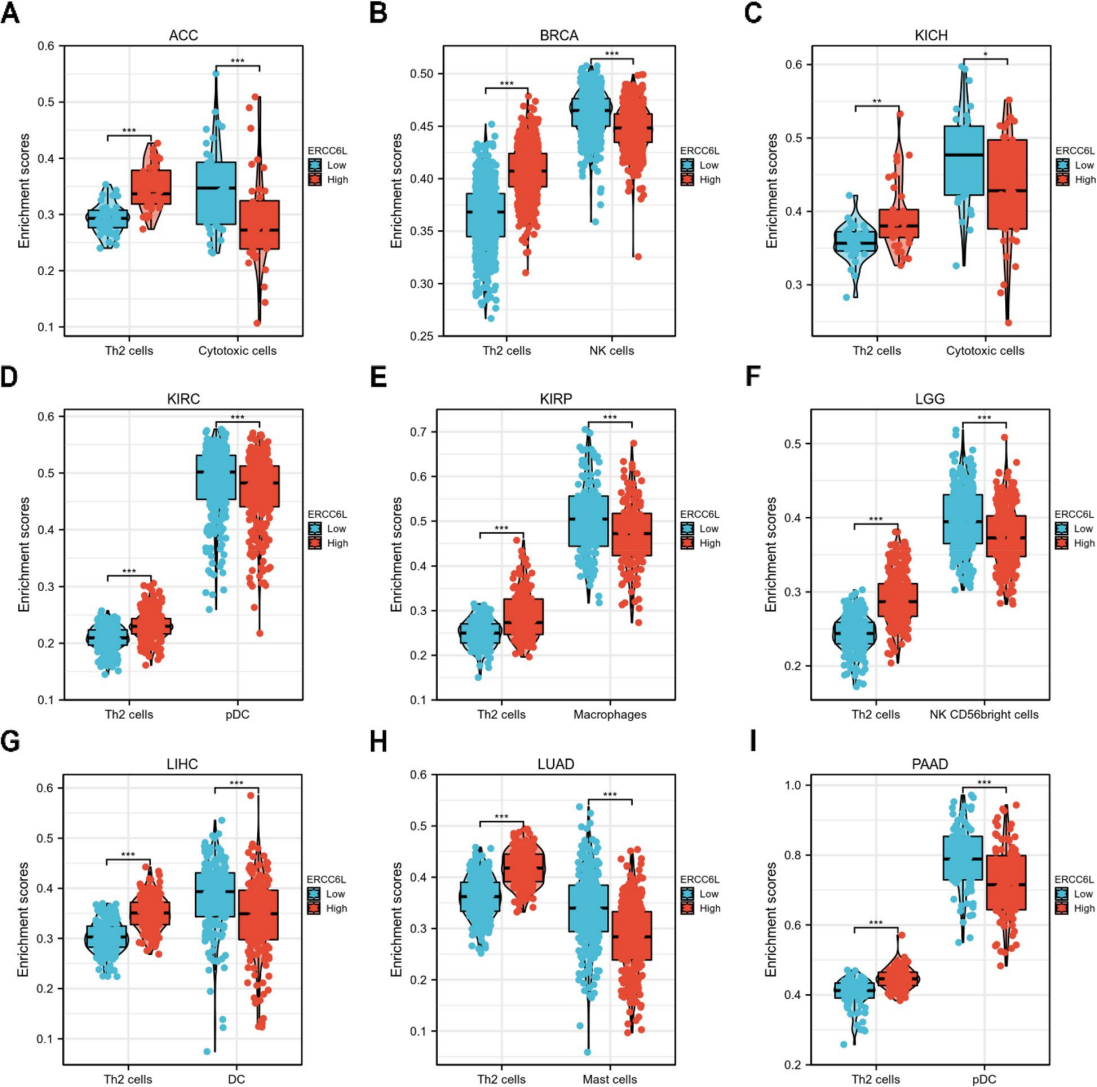
Analysis of immune cell infiltration in cancer patients grouped by ERCC6L mRNA expression levels. **A–I** Differential analysis of immune cell infiltration in the indicated cancer types, classified based on ERCC6L expression levels

To determine if ERCC6L is involved in immune cell infiltration, we quantified tumor purity in patients from each of the nine cancer categories. As displayed in Figs. S21A–I, a comprehensive analysis of the correlation between ERCC6L levels and infiltration of 24 immune cell types was conducted. We then identified the infiltrated immune cell types most positively or negatively associated with ERCC6L levels (Figs. 10A–I). Th2 cell infiltration positively correlated with ERCC6L expression levels across all studied cancer types. In general, immune cell infiltration was negatively associated with the ERCC6L levels, with various cell types being enriched in certain cancer types. For example, cytotoxic T cells were enriched in ACC and KICH, natural killer cells in BRCA and LGG, plasmacytoid dendritic cells in KIRC and PAAD, macrophages in KIRP, and mast cells in LUAD (Figs. 10A–I). In addition, the differential enrichment scores between the ERCC6L-low or -high groups were significantly altered, and the trends reflected the correlations depicted in Fig. 10 (Figs. 11A–I). Moreover, the StromalScore, ImmuneScore, and ESTIMATEScore analyses were performed, and significant correlations between ERCC6L and immune cell infiltration were mainly observed in BRCA, KIRC, and LUAD (Fig. S22). In conclusion, ERCC6L expression levels are strongly associated with the infiltration of various immune cells. Moreover, the types of infiltrating immune cells vary depending on the cancer type.

### Somatic mutation and drug sensitivity analysis of ERCC6L

**Fig. 12:**
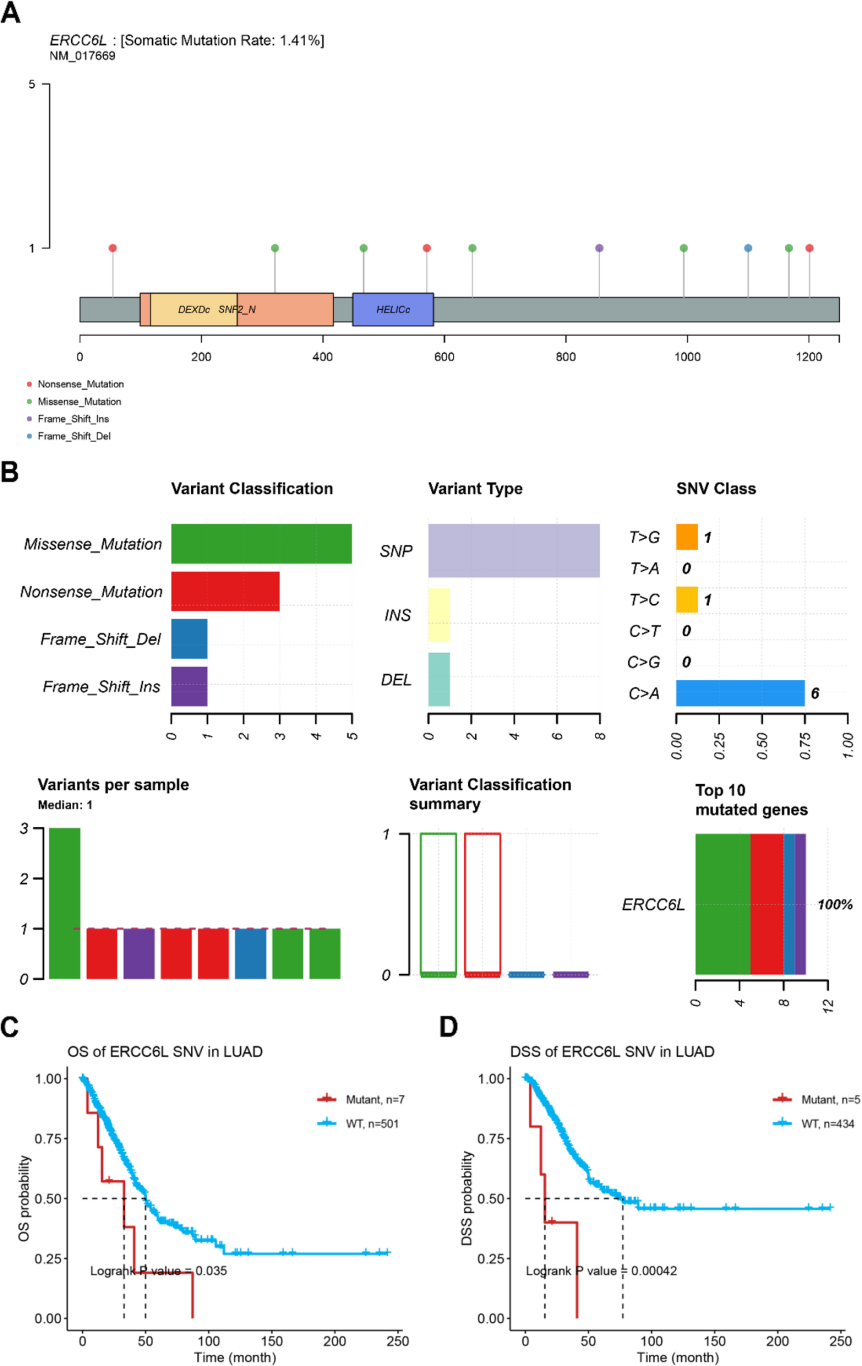
Analysis of somatic ERCC6L mutations. **A** Schematic showing the most common somatic mutation sites in ERCC6L gene. **B** Classification and frequency of ERCC6L gene variants. **C**, **D** Overall survival (OS, **C**) and disease-specific survival (DSS, **D**) analysis of patients with (mutant) or without (WT) an ERCC6L single-nucleotide variant

Somatic gene mutations contribute to cancer initiation in some cases [14]; therefore, we investigated the presence of somatic ERCC6L mutations in nine cancer types. The frequency of somatic mutations, including nonsense mutations, missense mutations, frameshift insertions, and frameshift deletions, resulting in single-nucleotide variants (SNVs), insertions, or deletions in the ERCC6L gene was 1.41% (Figs. 12A and B). Most somatic mutations were missense mutations, and C > A was the most prevalent class of SNV in the nine cancer types and BRCA when analyzed independently (Figs. 12B, S23, and S24). As indicated by the heatmap in Fig. S25, the SNV frequencies were markedly higher in LUAD and BRCA (8%) than in the other cancer types. Furthermore, significant decreases in patient OS and DSS rates were observed in LUAD patients with ERCC6L SNVs (Figs. 12C and D). In addition, by integrating data from the Cancer Therapeutics Response Portal and the Genomics of Drug Sensitivity in Cancer project, we identified correlations between ERCC6L mRNA expression levels and sensitivity to cancer therapeutic drugs (Figs. S26A and B).

## Discussion

Multiple disorders have been linked to ERCC6L protein dysregulation, which belongs to the ATPase family and is associated with the SWI/SWF complex [15]. ERCC6L expression levels are greatly alleviated in the neural tubes and hearts of mice with fetal alcohol syndrome [16]. Recently, it has been revealed that ERCC6L expression levels are highly linked with various cancer types. Liu et al. demonstrated that ERCC6L is commonly overexpressed in BRCA samples and loss-of-function analyses suggested that ERCC6L depletion decreases cell viability by inhibiting cell proliferation and augmenting apoptosis [17]. In addition, high ERCC6L expression levels have been identified as a prognostic marker for poor survival of HCC patients [18]. Like its effects on BRCA cells, ERCC6L potentiates the growth of HCC cells, and as downstream effectors of ERCC6L, the pro-cancerous pathways; PI3K/AKT and NF-κB, are activated [18, 19]. Moreover, the tumor-promoting functions of ERCC6L have been confirmed in renal cell carcinoma, gastric cancer, and colorectal cancer [5, 20, 21]. However, a previous study showed that ERCC6L is highly expressed in 12 solid cancer types by mining transcriptome data [4]. However, a comprehensive pan-cancer investigation is required to identify the expression pattern of ERCC6L in multiple cancer types and to determine the association between ERCC6L expression level and patient survival.

To thoroughly understand the role of ERCC6L in cancer, we identified a set of its co-expressed genes and performed GO and KEGG analyses. As expected, the biological processes associated with these co-expressed genes were enriched in nuclear and chromosomal actions, ATPase activity, and DNA modulation, which is in line with the previously reported functions of ERCC6L [15]. Cell cycle and DNA-related pathways, such as p53 and FoxO, were the most enriched processes and pathways in the ERCC6L-coexpressed gene set, as determined by KEGG analysis. These results strongly support the cell mentioned above the growth-enhancing effect of ERCC6L in cancer. However, to obtain a complete picture of the genes or pathways affected by ERCC6L, it is necessary to perform the transcriptome profiling of cancer cells with dysregulated ERCC6L expression, and unbiased pathway enrichment analysis validate the bioinformatics results. Furthermore, gene set enrichment analysis and experimental evaluation were employed to confirm these results.

We systematically analyzed the expression levels of ERCC6L in a large panel of diverse cancer types and confirmed that ERCC6L was generally overexpressed in tumor tissues in most cases (Fig. 1). Subsequent survival analysis demonstrated that ERCC6L served as an independent diagnostic and prognostic marker for poor outcomes in most cancer types (Figs. 2, 3, 4). Furthermore, the ERCC6L expression level was markedly higher in tumor samples with higher clinical grades. It was an indicator of poor survival rates regardless of the clinical grade (Figs. 5 and 6). This finding was unexpected, considering that most prognostic markers are only effective in subtype-specific patient populations. Thus, as an effective biomarker, ERCC6L may serve as an adjuvant marker in combination with other prognostic markers or clinical parameters to aid in providing a more accurate patient prognosis with cancers subdivided into various pathological classes.

We have shown negative correlations between ERCC6L mRNA expression levels and its promoter methylation in most cancers, excluding PAAD (Fig. S20). We assumed that other regulatory mechanisms besides promoter methylation modulated ERCC6L mRNA expression. Thus, more investigations are required to be performed on PAAD patients. The ERCC6L promoter was hypomethylated in HCC, which may result in increased ERCC6L expression levels in HCC tissues than in normal compartments [22]. Consistent with this prior finding, our analysis of BRCA patients depicted that ERCC6L levels were inversely correlated with promoter methylation levels. Notably, higher methylation levels of the ERCC6L promoter resulted in favorable outcomes for BRCA patients. This observation suggested that gene modulation at the transcriptional level is a key process in regulating ERCC6L expression. Deciphering the methods by which ERCC6L promoter activity is regulated may offer a greater understanding of the complexity of ERCC6L dysregulation in cancer. Moreover, identifying the DNA elements responsible for the demethylation of ERCC6L could lead to the development of novel therapeutic strategies for targeting ERCC6L in cancer.

The tumor microenvironment modulates cancer progression, especially the infiltration of specific immune cells [23]. Depending on their nature, immune cells may contribute to the resistance of tumor cells to therapeutic drugs or kill tumor cells. Thus, determining the types and percentage of infiltrating immune cells in the surrounding tumor microenvironment helps to determine which clinical interventions can be exploited to inhibit cancer progression. Our analysis of immune cell infiltration in different cancer types demonstrated that Th2 cells were the most common class of infiltrating immune cells positively correlated with ERCC6L expression levels. However, it has been shown that Th2 cells inhibit the growth of tumor cells [24] and hence participate in tumor immune surveillance. Therefore, evidence from other models, such as mouse xenograft models with ERCC6L-depleted cancer cells and the subsequent flow cytometric analysis of immune cell percentages, can be utilized to elucidate better the correlations between ERCC6L expression levels and immune cell infiltration.

The present study had several limitations that should be noted. For some rare cancer types, like CHOL and READ, the patient sample sizes were limited, which may have resulted in inaccurate analyses. In addition, more databases with larger sample sizes should be included in future studies to obtain a better overview and validate our results. We only included ERCC6L mRNA levels in our study because transcriptome data are more readily available on open access platforms. As the ERCC6L protein affects cancer progression, its expression pattern and correlation with patient prognosis require further examination in patient samples, for example, by performing immunohistochemistry on tissue microarrays containing normal and cancer samples or tumor samples from a diverse range of clinical stages. Moreover, our pan-cancer analysis results were derived from bioinformatics approaches. Additional experiential evidence is needed to determine the effects of inhibiting ERCC6L on the progression of cancers of interest.

## Supplementary Information

**Additional file 1.** Supplementary figures.

## Data Availability

The authors declare that all data supporting the findings of this study are available within the article and its supplementary information files. Data available in a public repository that issues datasets with https://datadryad.org/stash/share/83aq_Hqq05UBtlltbe4riD8BXMqSijCVOQs7MWuXBeg. These data include gene expression and clinically relevant data of ACC, BRCA, KICH, KIRC, KIRP, LGG, LIHC, PAAD, LUAD…, which can be downloaded with TCGA (https://portal.gdc.cancer.gov/).
