# Supplementary material for: A pan-cancer analysis of the oncogenic role of ERCC6L
Source: BMC Cancer. 2022 Dec 22;22:1347. doi: 10.1186/s12885-022-10452-3 (PMC9773625; doi:10.1186/s12885-022-10452-3)
Supplement: Supplementary file 1 — Additional file 1. Supplementary figures. [file 12885_2022_10452_MOESM1_ESM.docx]

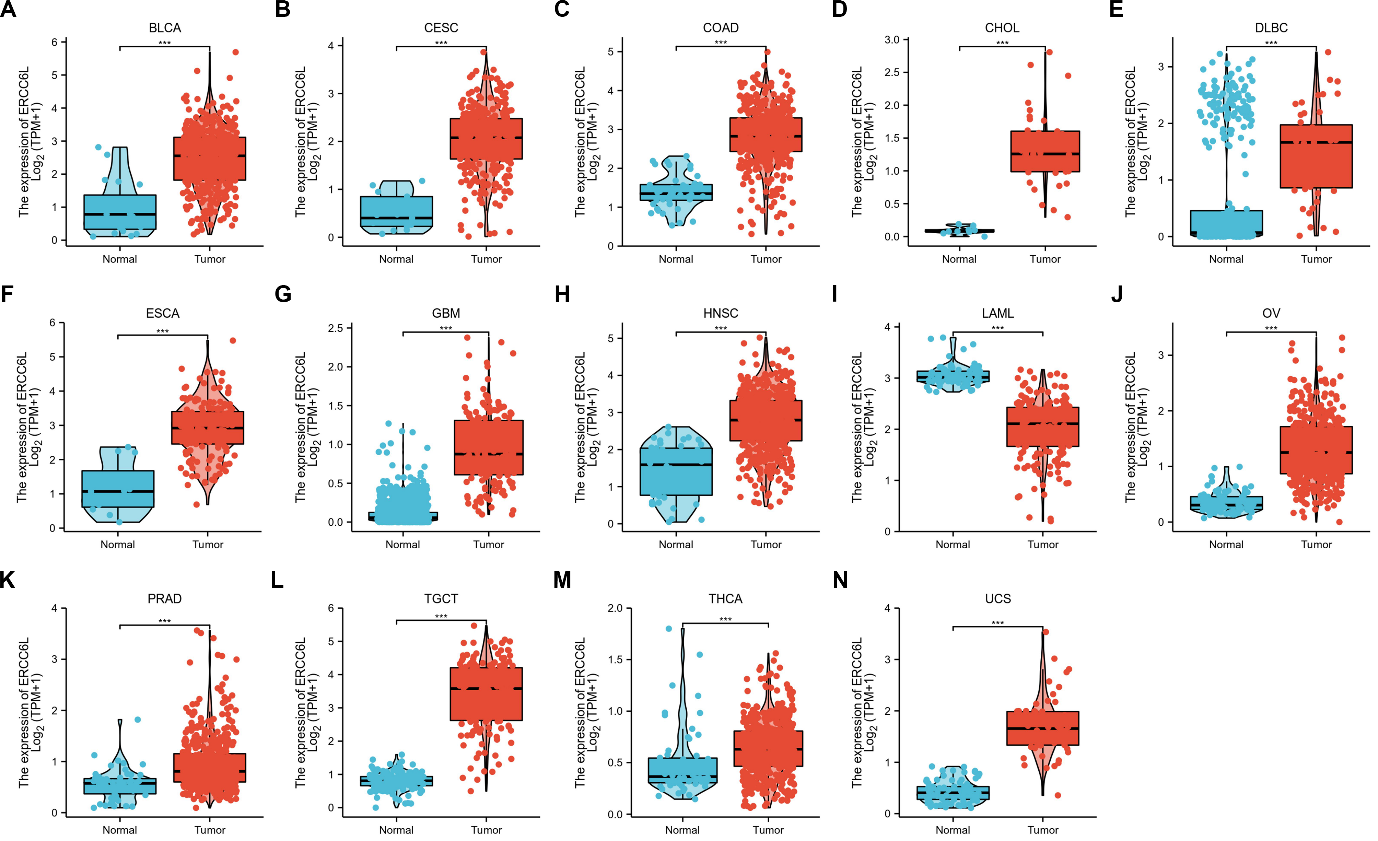


**Fig. S1 Expression of ERCC6L in diverse cancer types.**

Differential expression analysis of ERCC6L in multiple cancers including BLCA (A, CESC (B), COAD (C), CHOL (D), DLBC (E), ESCA (F), GBM (G), HNSC (H), LAML (I), OV (J), PRAD (K), TGCT (L), THCA (M) and UCS (N). The data were derived from TCGA database and statistical analyses were performed between the levels of ERCC6L in normal and tumor samples.


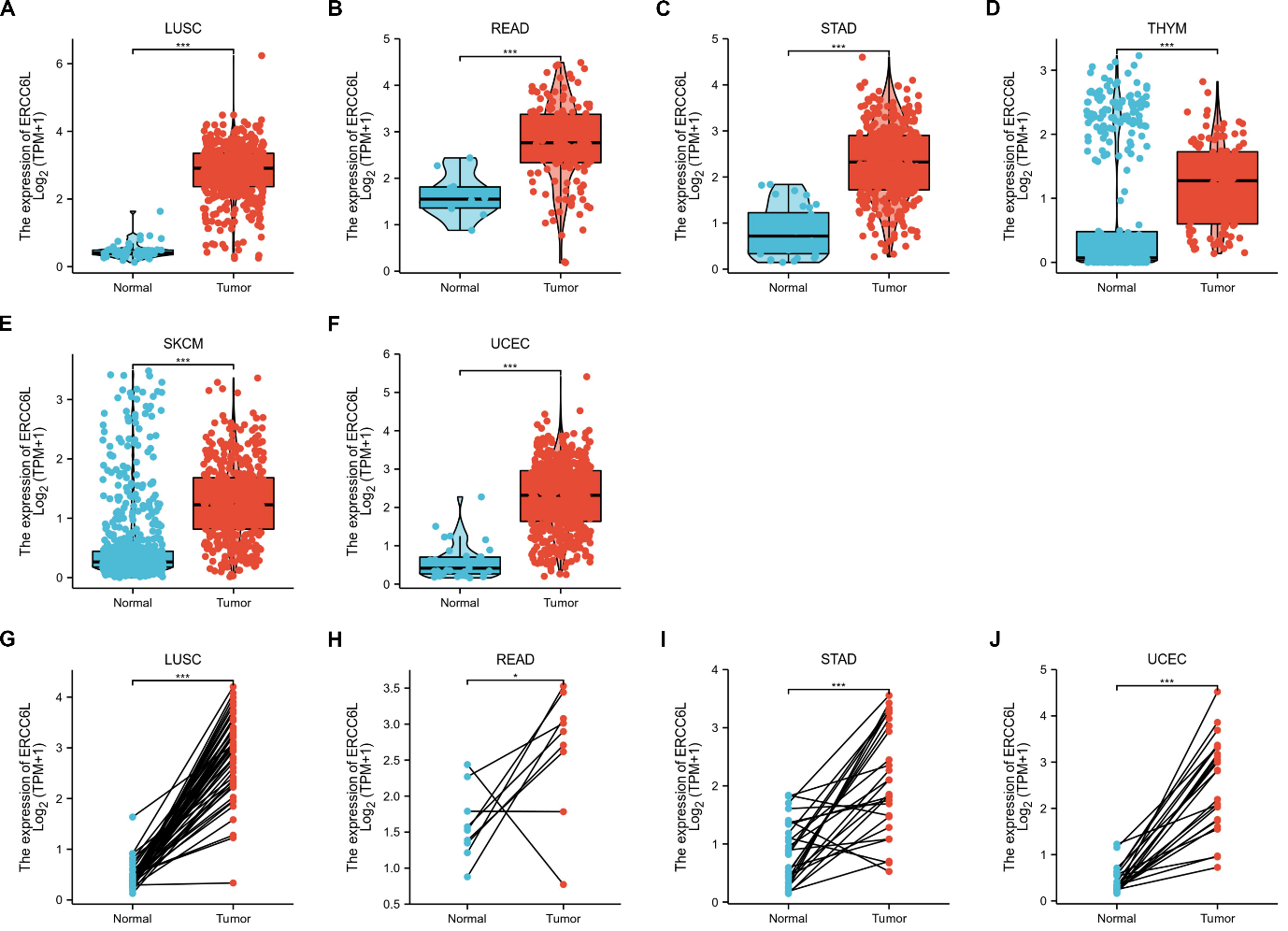


**Fig. S2 Expression of ERCC6L in various cancer types.**

(A-F) Differential expression analysis of ERCC6L in multiple cancers including LUSC (A), READ (B), STAD (C), THYM (D), SKCM (E) and UCEC (F). The data were derived from the TCGA database and statistical analyses were performed between the levels of ERCC6L in normal and tumor samples. Differential expression analysis of ERCC6L in paired tissues from multiple cancers including LUSC (G), READ (H), STAD (I) and UCEC (J). The data were derived from the TCGA database and statistical analyses were performed between the levels of ERCC6L in normal and tumor samples.


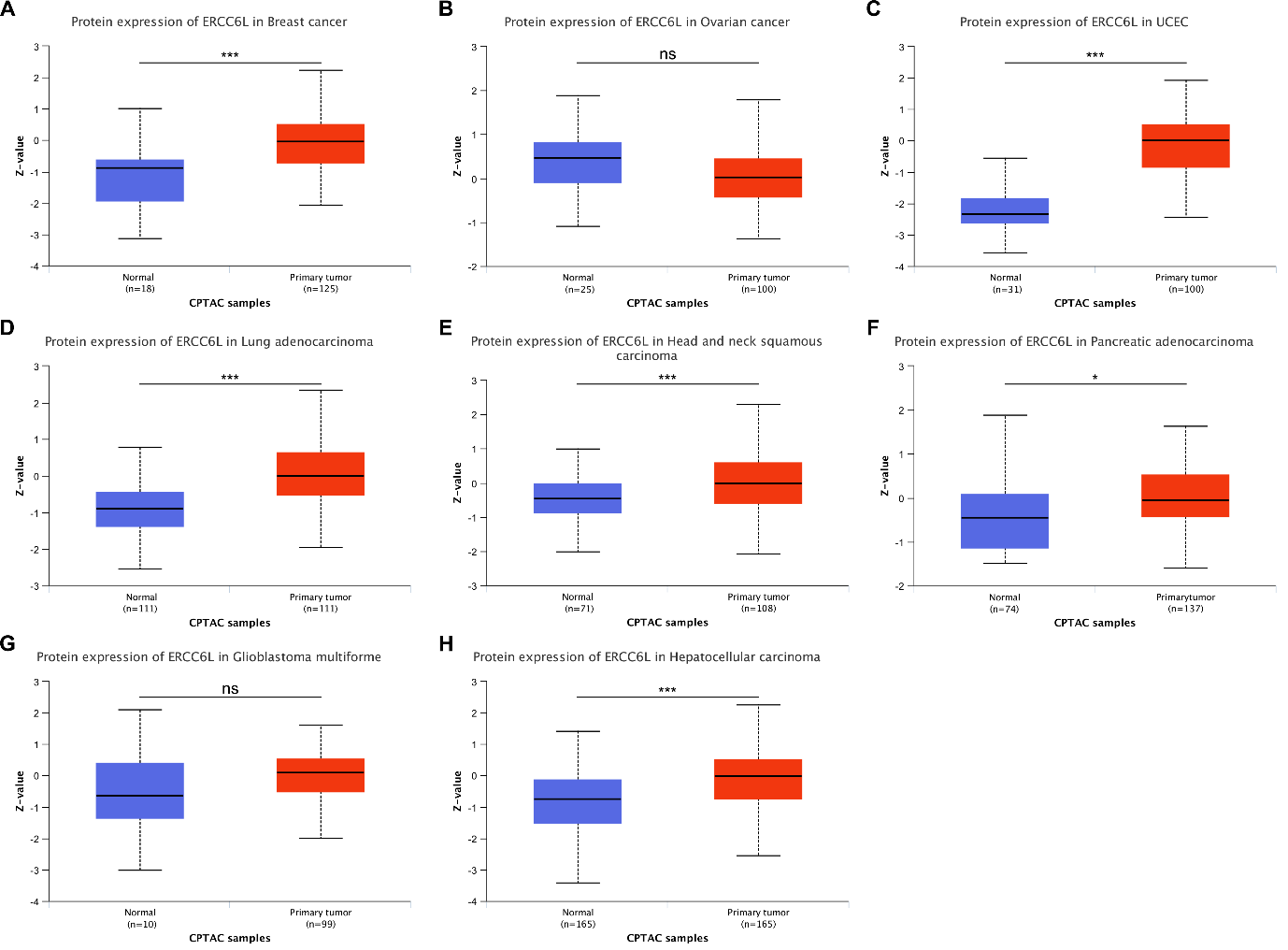


**Fig. S3 Protein expression of ERCC6L in paired samples from patients with various cancers.**

(A-F) Differential expression analysis of ERCC6L in paired tissues from multiple cancers including breast cancer (A), ovarian cancer (B), UCEC (C), lung adenocarcinoma (D), head and neck squamous carcinoma (E), pancreatic carcinoma (F), glioblastoma (G) and hepatocellular carcinoma (H). The data were derived from the UALCAN database and statistical analyses were performed between the levels of ERCC6L in normal and tumor samples.


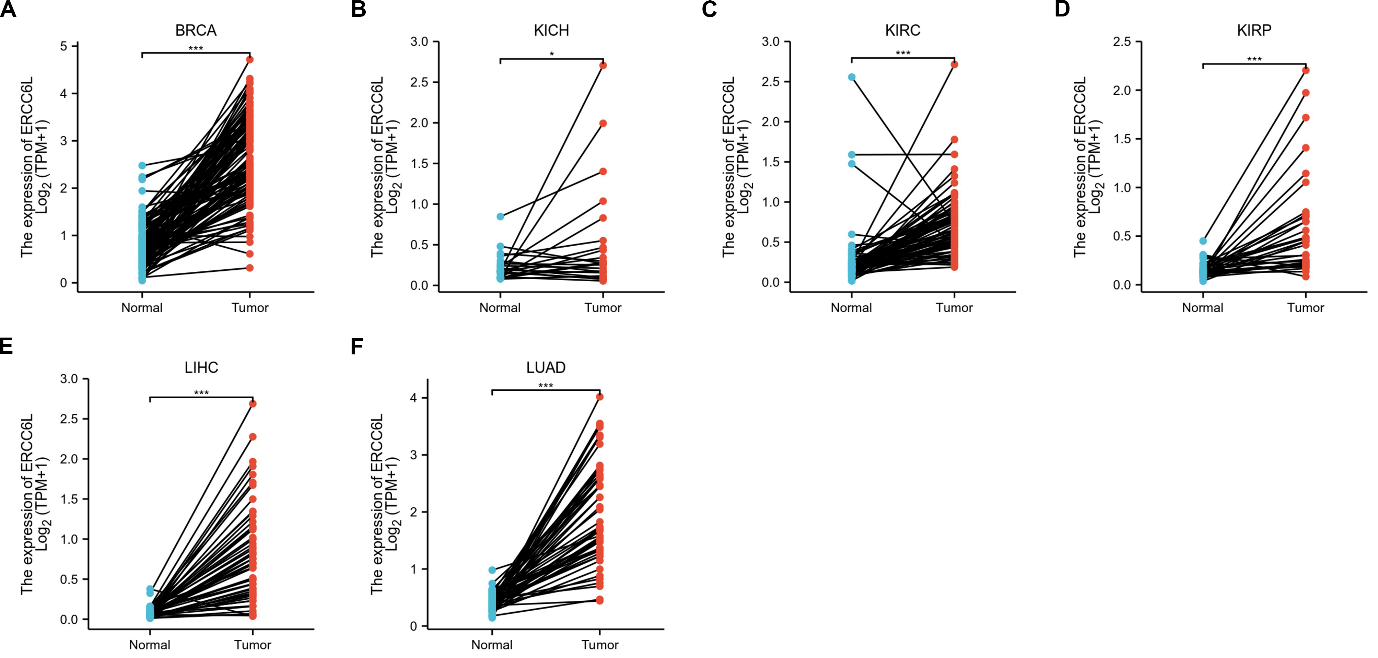


**Fig. S4 Expression of ERCC6L in paired samples from patients with various cancers.**

(A-F) Differential expression analysis of ERCC6L in paired tissues from multiple cancers including BRCA (A), KICH (B), KIRC (C), KIRP (D), LIHC (E) and LUAD (F). The data were derived from the TCGA database and statistical analyses were performed between the levels of ERCC6L in normal and tumor samples.

**
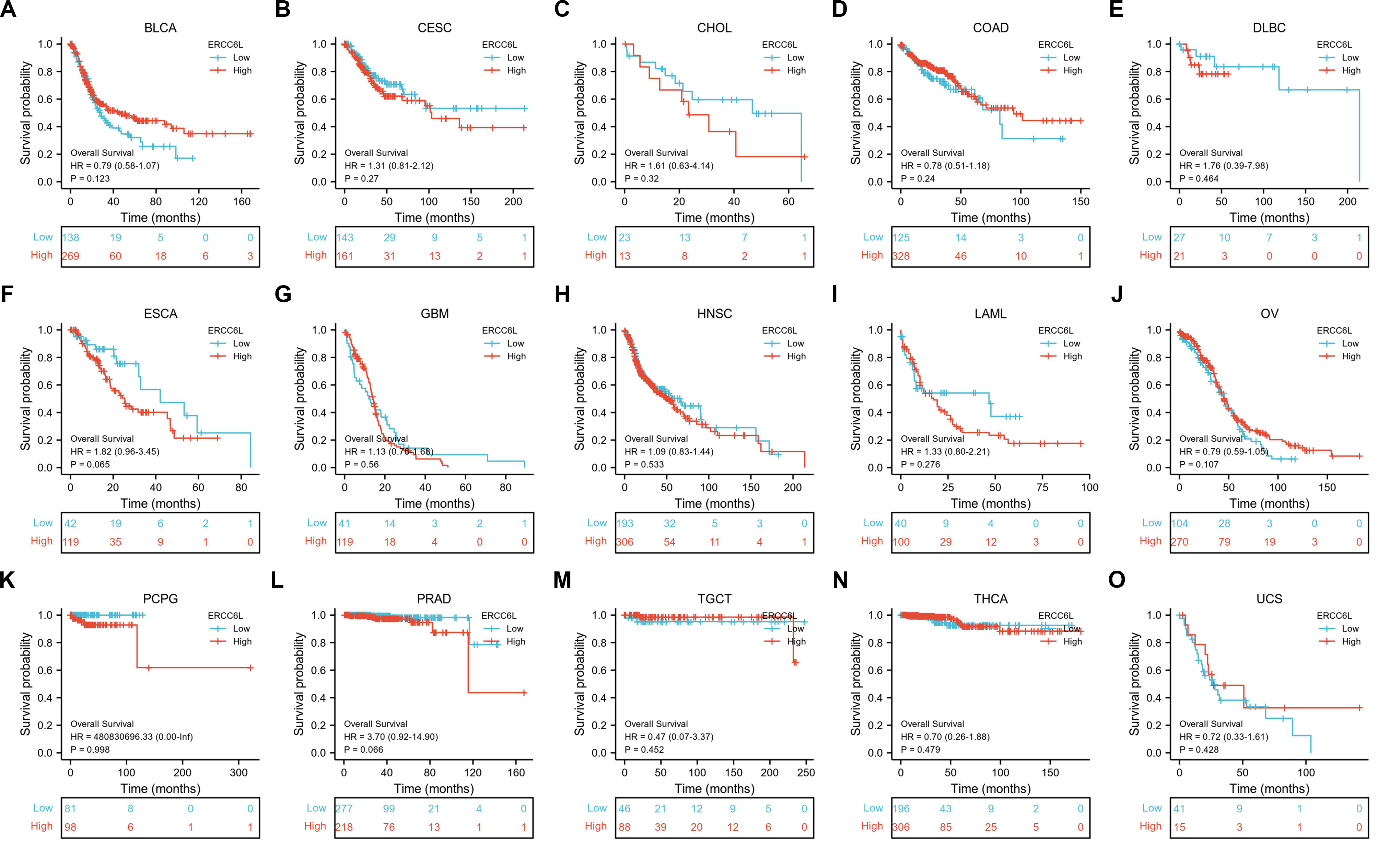
**

**Fig. S5 The association between ERCC6L expression and overall survival of cancer patients.**

(A-O) Overall survival analysis of cancer patients stratified by ERCC6L expression based on data from TCGA platform. The patients were diagnosed as BLCA (A), CESC (B), CHOL (C), COAD (D), DLBC (E), ESCA (F), GBM (G), HNSC (H), LAML (I), OV (J), PCPG (K), PRAD (L), TGCT (M), THCA (N) and UCS (O), respectively.

**
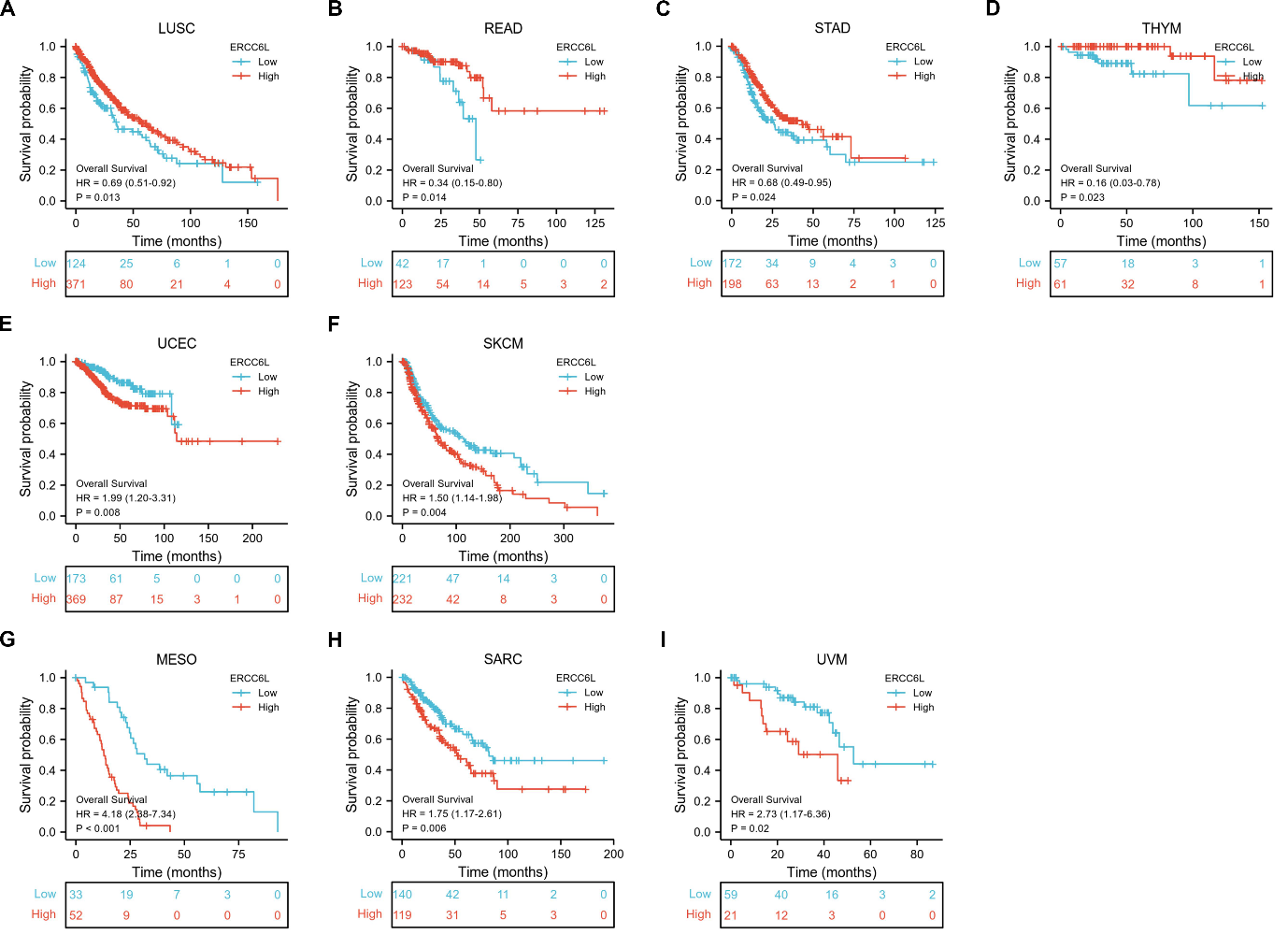
**

**Fig. S6 The association between ERCC6L expression and overall survival of cancer patients.**

(A-I) Overall survival analysis of cancer patients stratified by ERCC6L expression based on data from TCGA platform. The patients were diagnosed as LUSC (A), READ (B), STAD (C), THYM (D), UCEC (E), SKCM (F), MESO (G), SARC (H) and UVM (I), respectively.

**
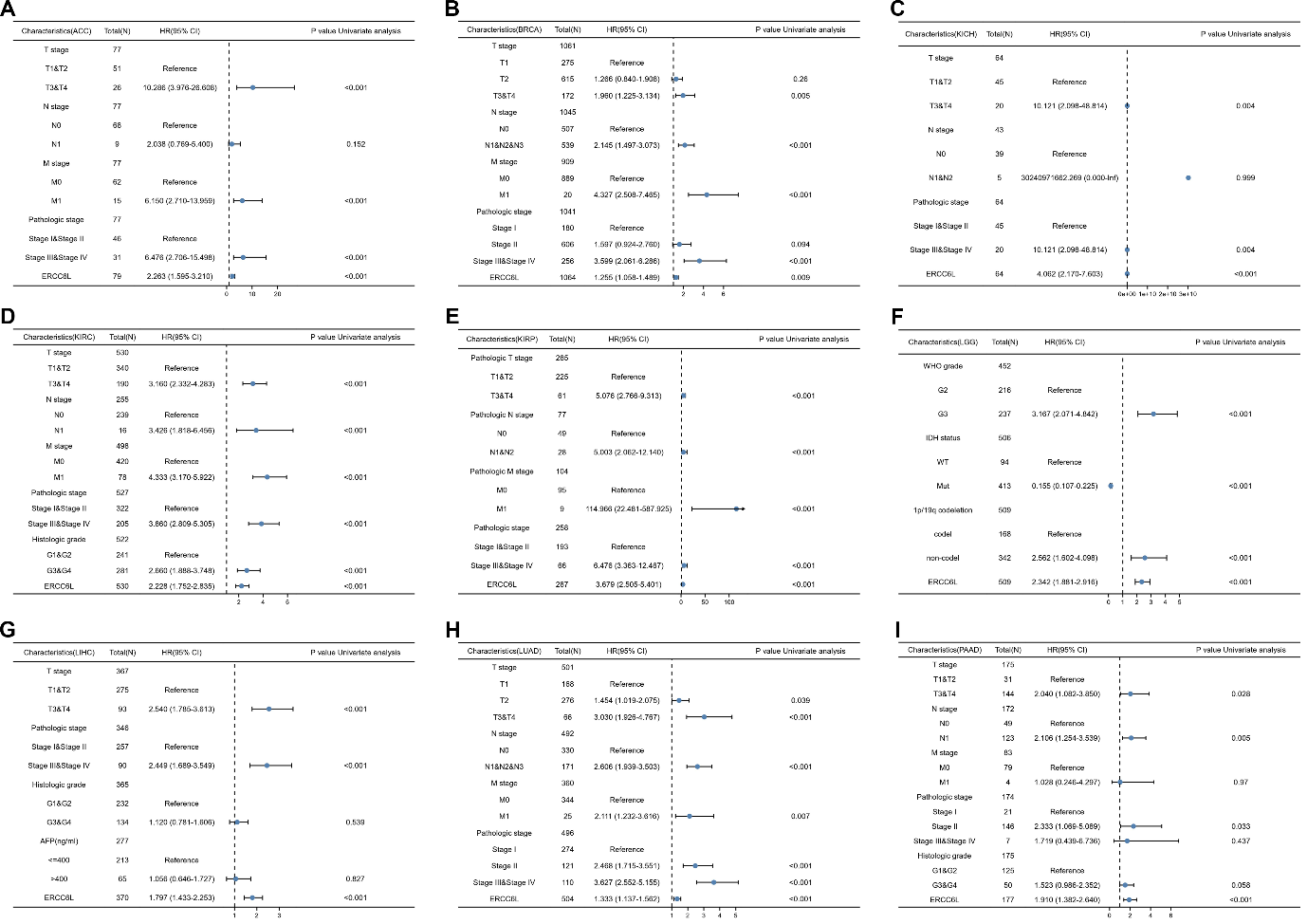
**

**Fig. S7 Univariable cox regression hazards analysis of overall survival in patients with suggested cancers, respectively.** The results were categorized as patients with ACC (A), BRCA (B), KICH (C), KIRC (D), KIRP (E), LGG (F), LIHC (G), LUAD (H) and PAAD (I), respectively.

**
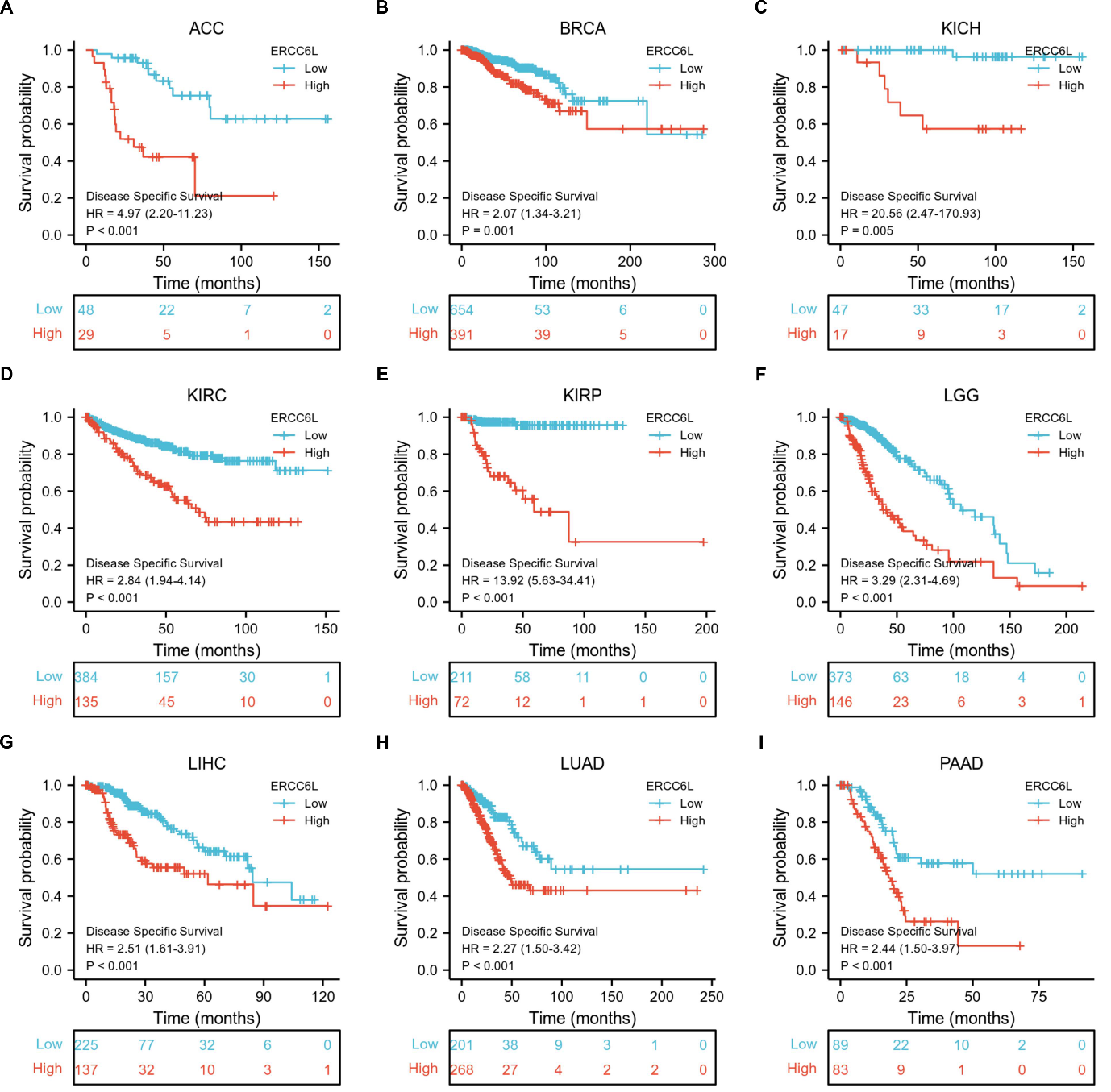
**

**Fig. S8 The association between ERCC6L expression and the disease specific survival of cancer patients.**

(A-O) Disease specific survival (DSS) analysis of cancer patients stratified by ERCC6L expression. The patients were diagnosed as ACC (A), BRCA (B), KICH (C), KIRC (D), KIRP (E), LGG (F), LIHC (G), LUAD (H) and PAAD (I), respectively.

**
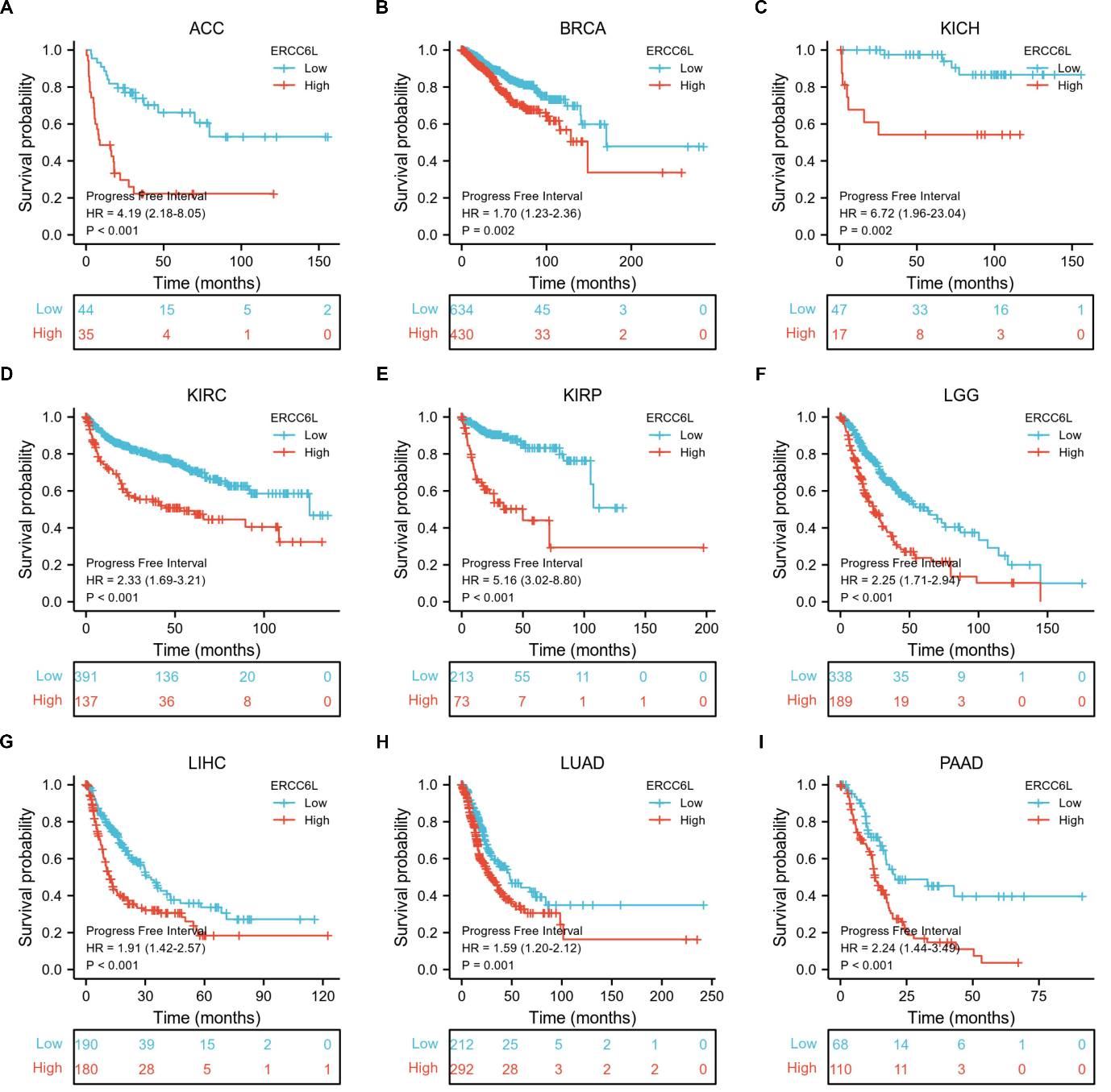
**

**Fig. S9 The association between ERCC6L expression and the progression free survival of cancer patients.**

(A-O) Progression free interval (PFI) analysis of cancer patients stratified by ERCC6L expression. The patients were diagnosed as ACC (A), BRCA (B), KICH (C), KIRC (D), KIRP (E), LGG (F), LIHC (G), LUAD (H) and PAAD (I), respectively.

**
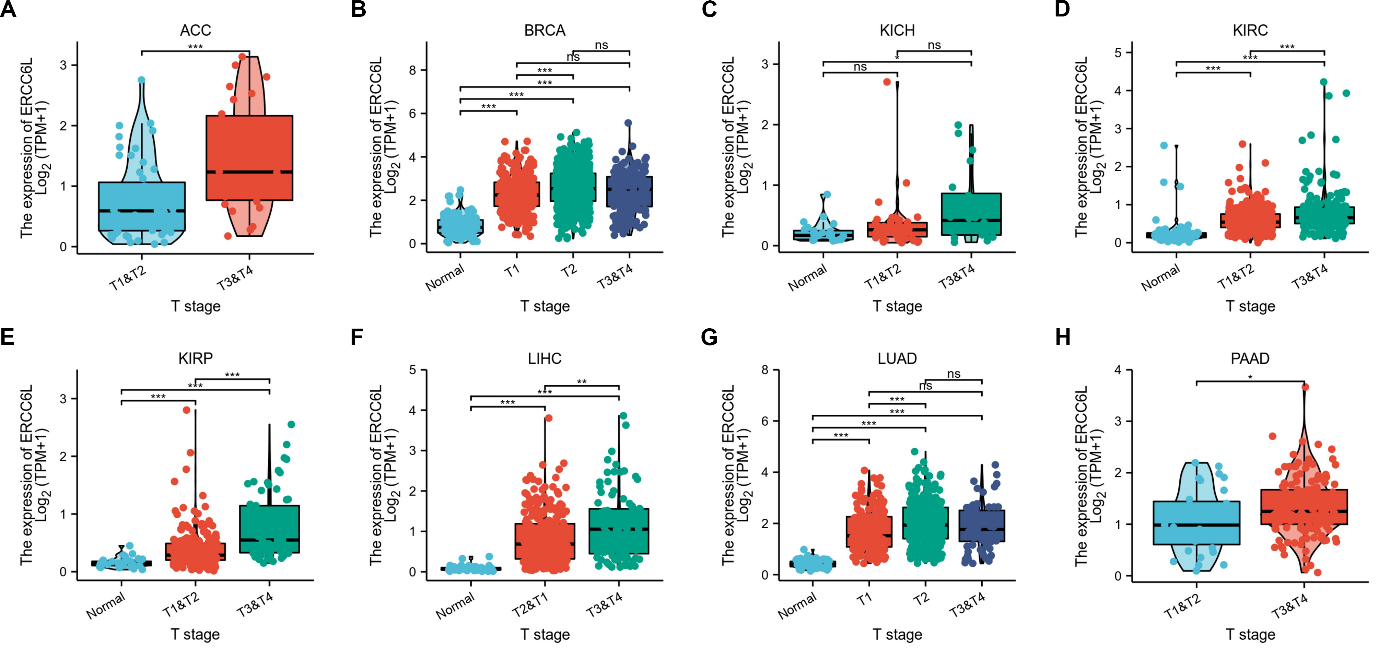
**

**Fig. S10 Expression of ERCC6L in cancer patients belonging to different clinical T stages.**

(A-H) Differential expression analysis of ERCC6L in diverse clinical T stages of multiple indicated cancers. The patients were diagnosed as ACC (A), BRCA (B), KICH (C), KIRC (D), KIRP (E), LIHC (F), LUAD (G) and PAAD (H), respectively. The results were analysed based on the data collected from TCGA dataset.

**
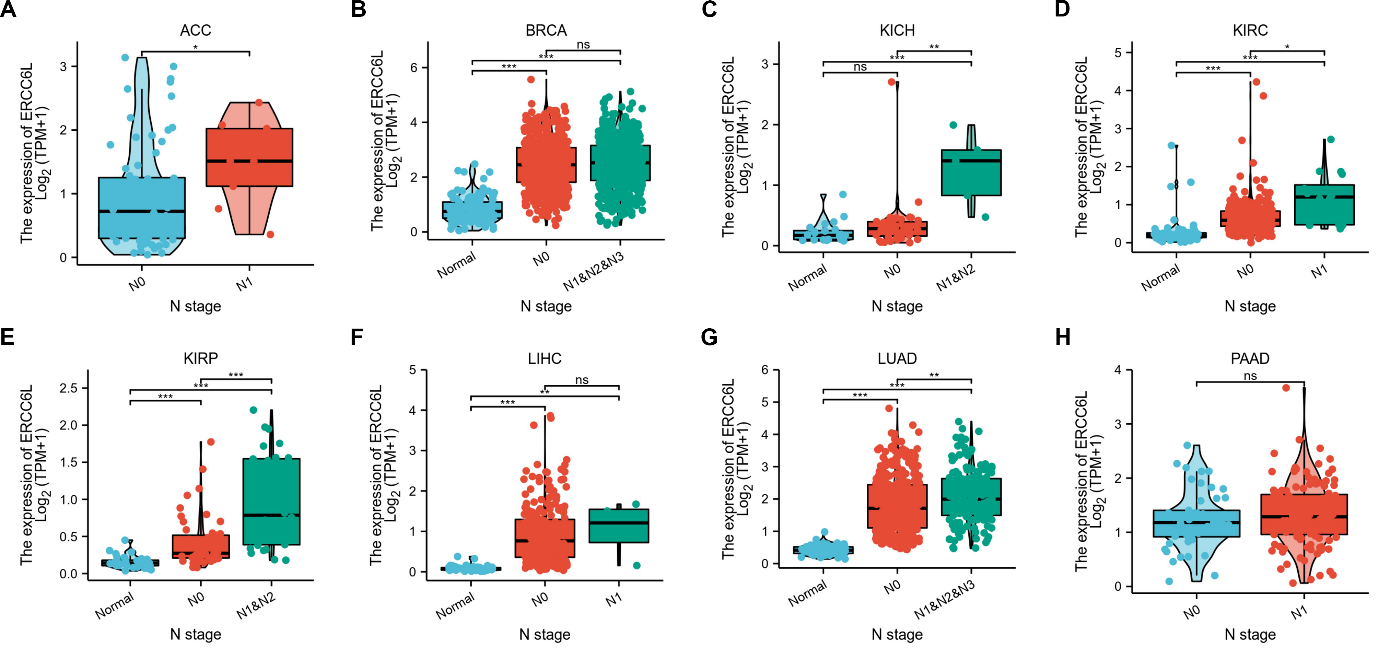
**

**Fig. S11 Expression of ERCC6L in cancer patients belonging to different clinical N stages.**

(A-H) Differential expression analysis of ERCC6L in diverse clinical N stages of multiple indicated cancers. The patients were diagnosed as ACC (A), BRCA (B), KICH (C), KIRC (D), KIRP (E), LIHC (F), LUAD (G) and PAAD (H), respectively. The results were analysed based on the data collected from the TCGA dataset.

**
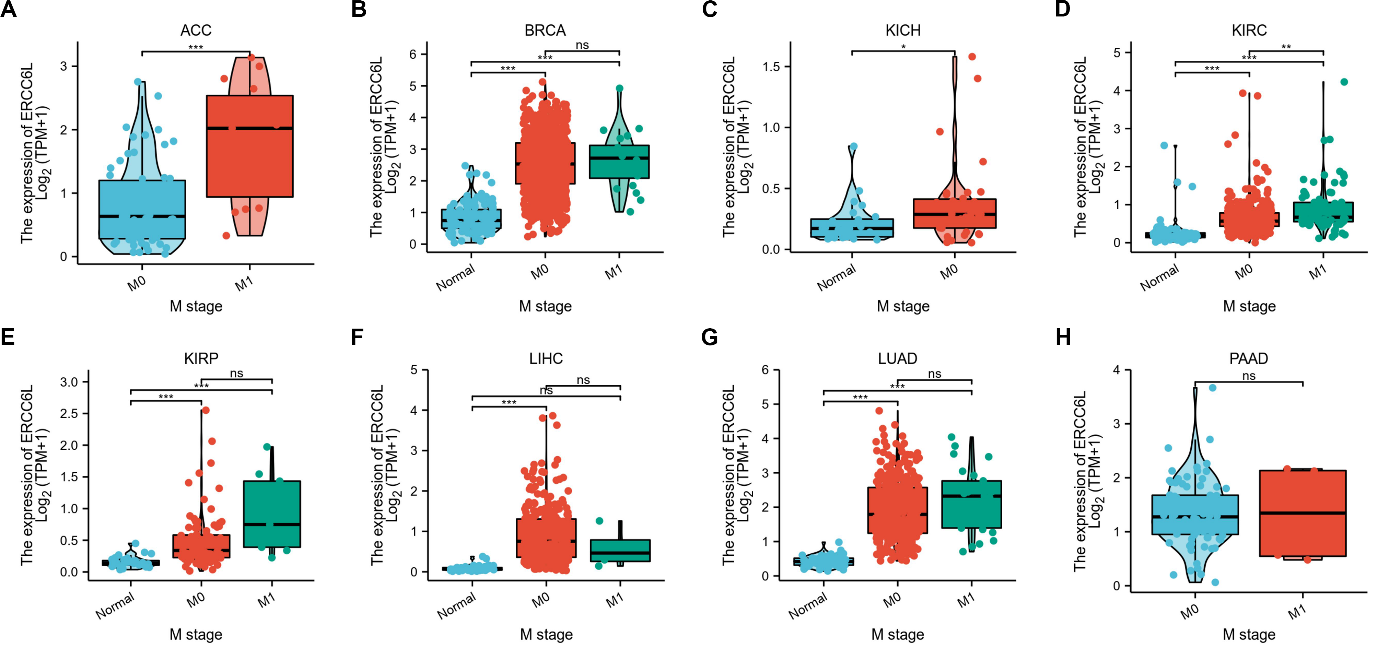
**

**Fig. S12 Expression of ERCC6L in cancer patients belonging to different clinical M stages.**

(A-H) Differential expression analysis of ERCC6L in diverse clinical M stages of multiple indicated cancers. The patients were diagnosed as ACC (A), BRCA (B), KICH (C), KIRC (D), KIRP (E), LIHC (F), LUAD (G) and PAAD (H), respectively. The results were analysed based on the data collected from the TCGA dataset.

**
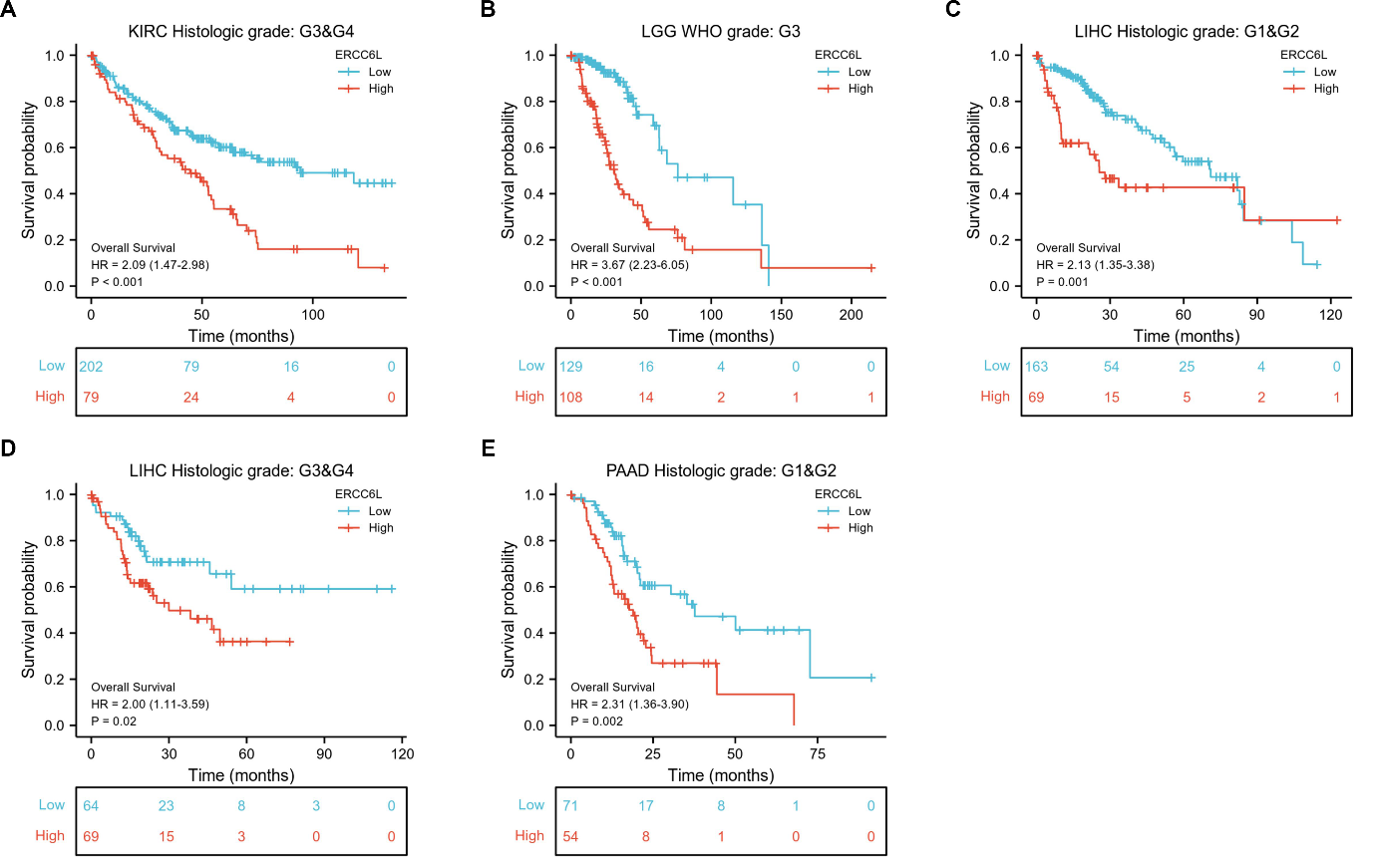
**

**Fig. S13 Survival analysis of patients in diverse clinical histological stages based on ERCC6L expression.**

(A-E) Overall survival analysis of cancer patients stratified by ERCC6L expression. The patients were classified into indicated clinical stages in different cancers.

**
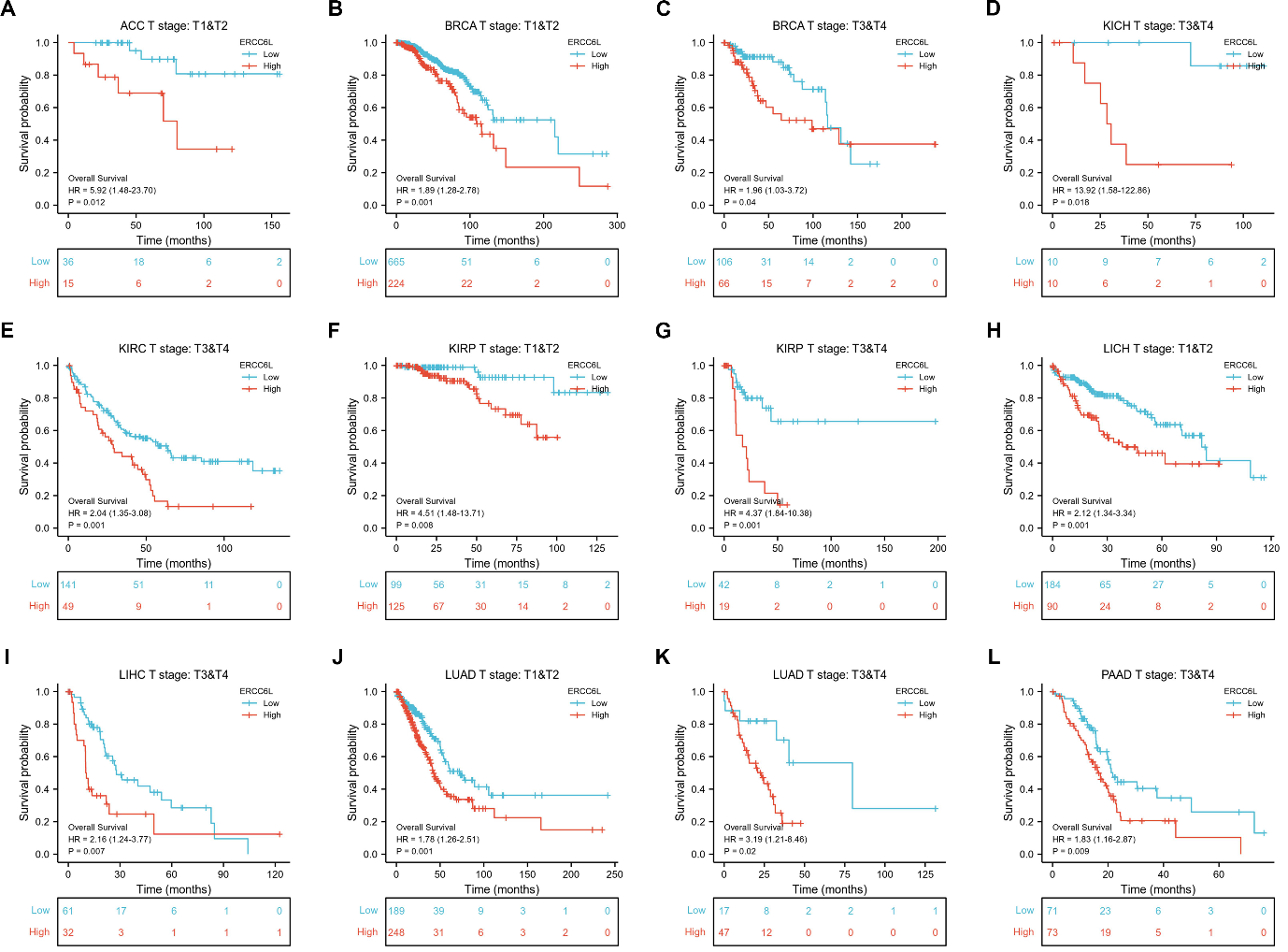
**

**Fig. S14 Survival analysis of patients in diverse clinical T stages based on ERCC6L expression.**

(A-E) Overall survival analysis of cancer patients stratified by ERCC6L expression. The patients were classified into indicated clinical stages in different cancers.

**
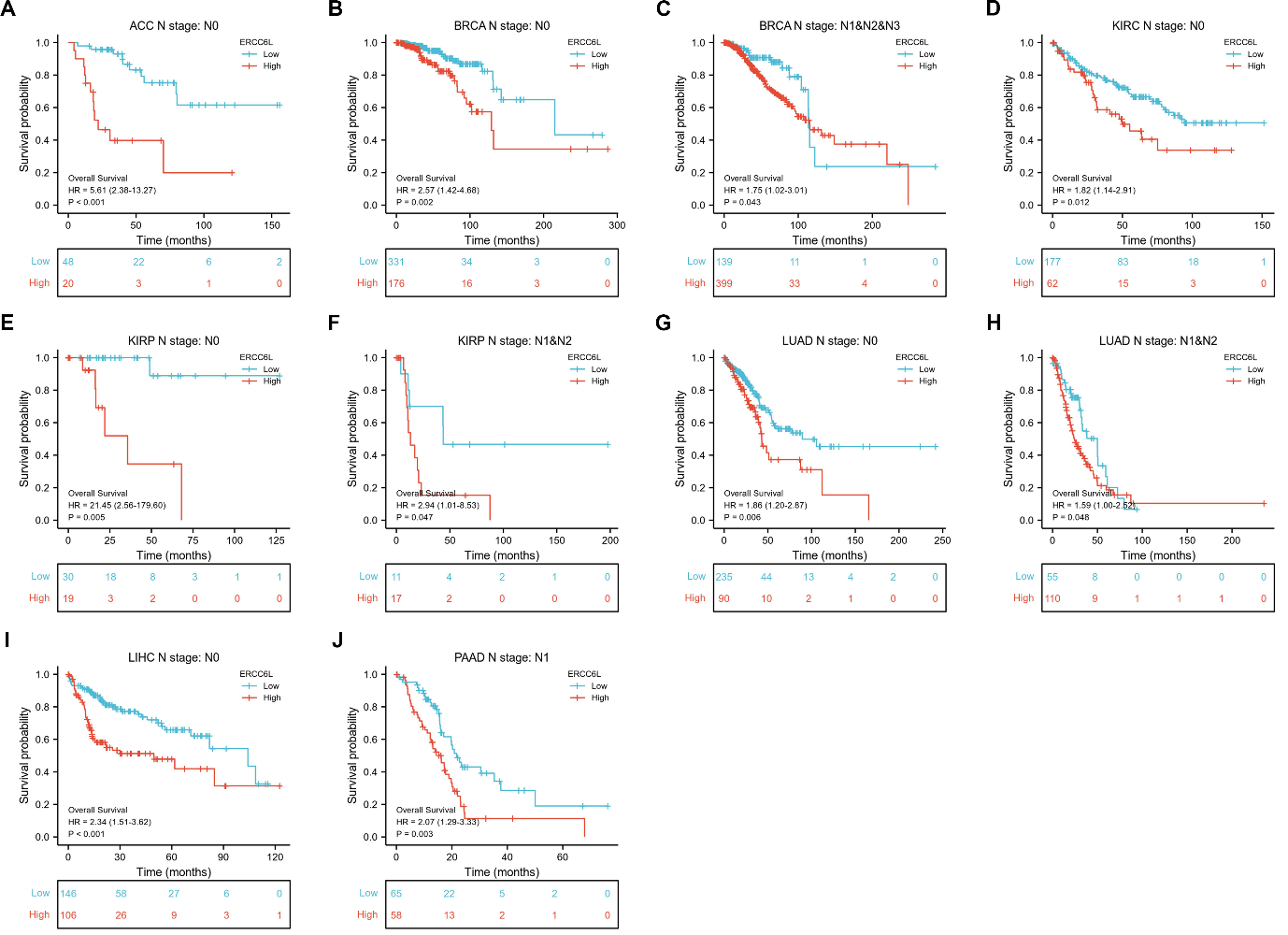
**

**Fig. S15 Survival analysis of patients in diverse clinical N stages based on ERCC6L expression.**

(A-E) Overall survival analysis of cancer patients stratified by ERCC6L expression. The patients were classified into indicated clinical stages in different cancers.

**
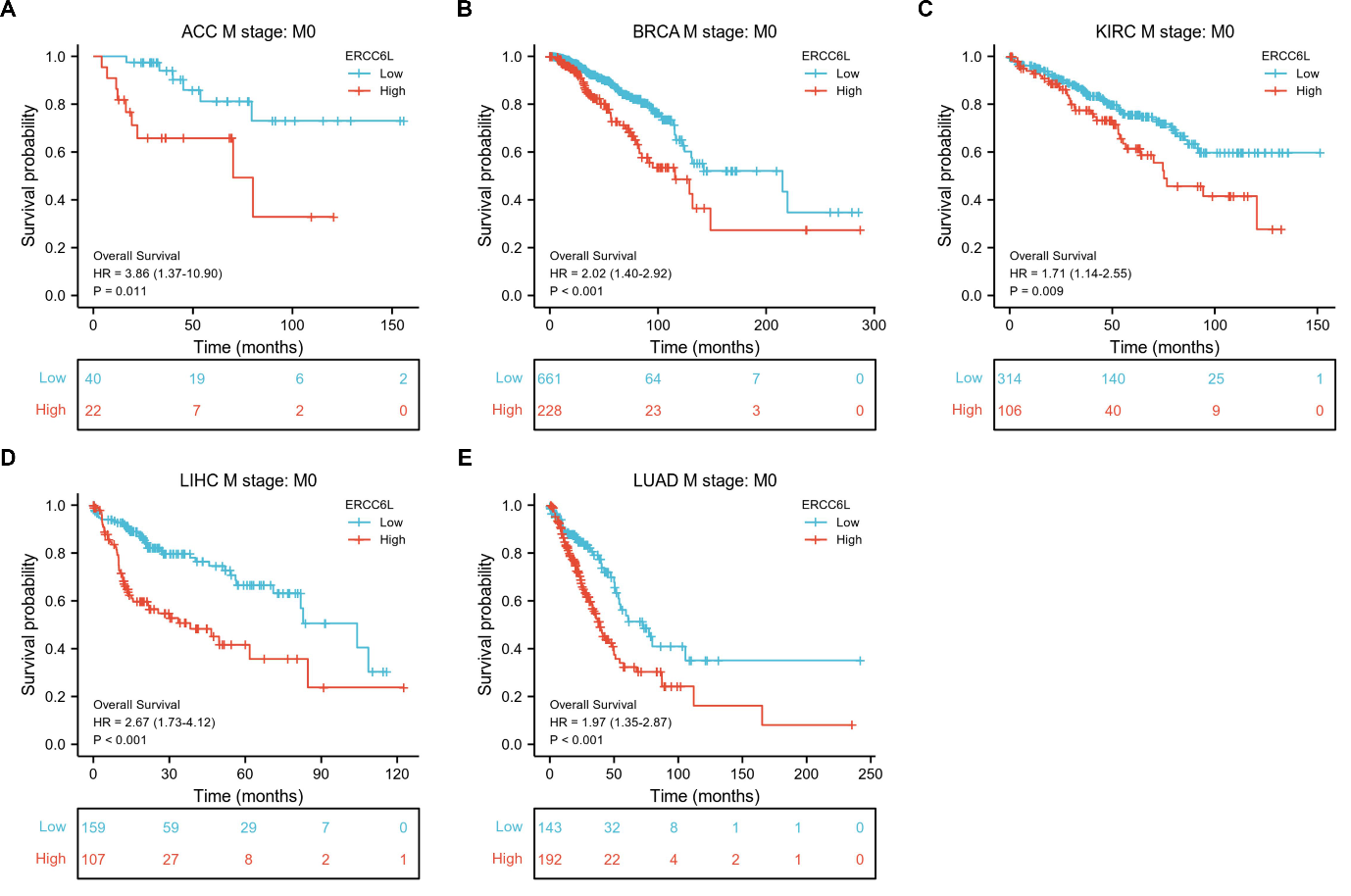
**

**Fig. S16 Survival analysis of patients in M0 stages based on ERCC6L expression.**

(A-E) Overall survival analysis of cancer patients stratified by ERCC6L expression. The patients were classified into indicated clinical stages in different cancers.

**
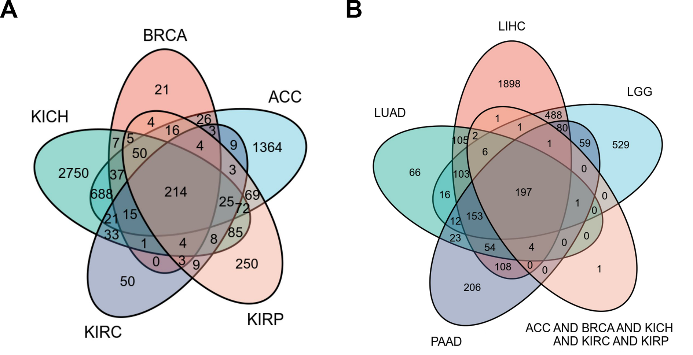
**

**Fig. S17 Enrichment of genes co-expressed with ERCC6L.**

(A) Venn diagram showing the genes co-expressed with ERCC6L in BRCA, KICH, KIRC, ACC and KIRP. (B) Venn diagram showing the genes co-expressed with ERCC6L in figure A dataset, LIHC, LUAD, PAAD and LGG.

**
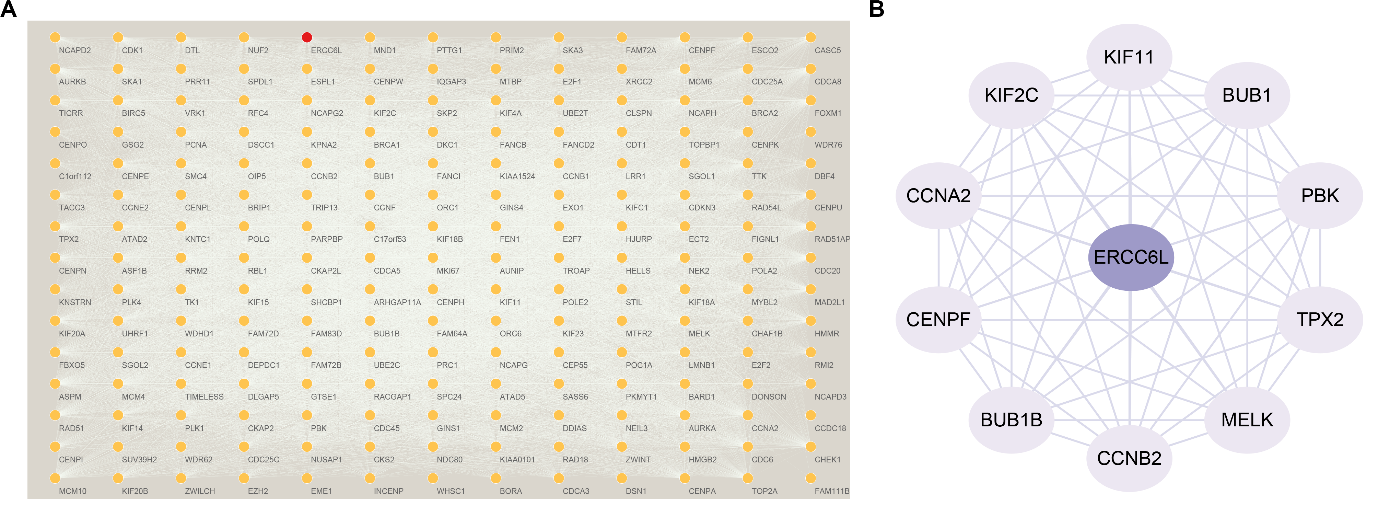
**

**Fig. S18 Network analysis of genes co-expressed with ERCC6L.**

Hub gene analysis among 197 ERCC6L co-expressed genes (A) and Protein-Protein interaction network among the top correlated genes (B).

**
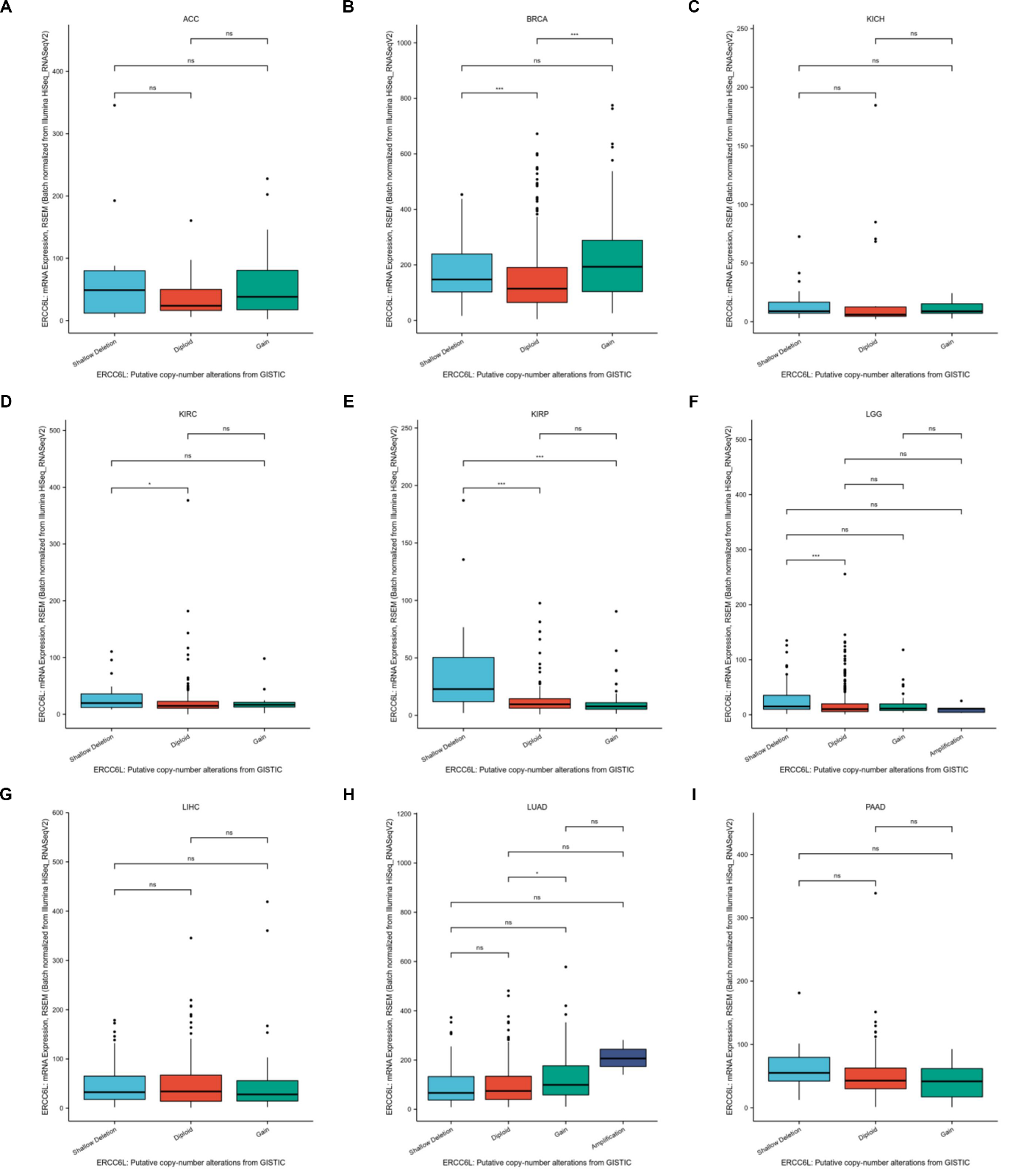
**

**Fig. S17 Correlation analysis of mRNA levels of ERCC6L and copy number variations in different cancer patients.**

(A-I) Differential comparison of the ERCC6L mRNA levels in various indicated putative copy-number alterations (CNV; shallow deletion, diploid, gain or amplification) in cancer patient samples from the cBioportal database.

**
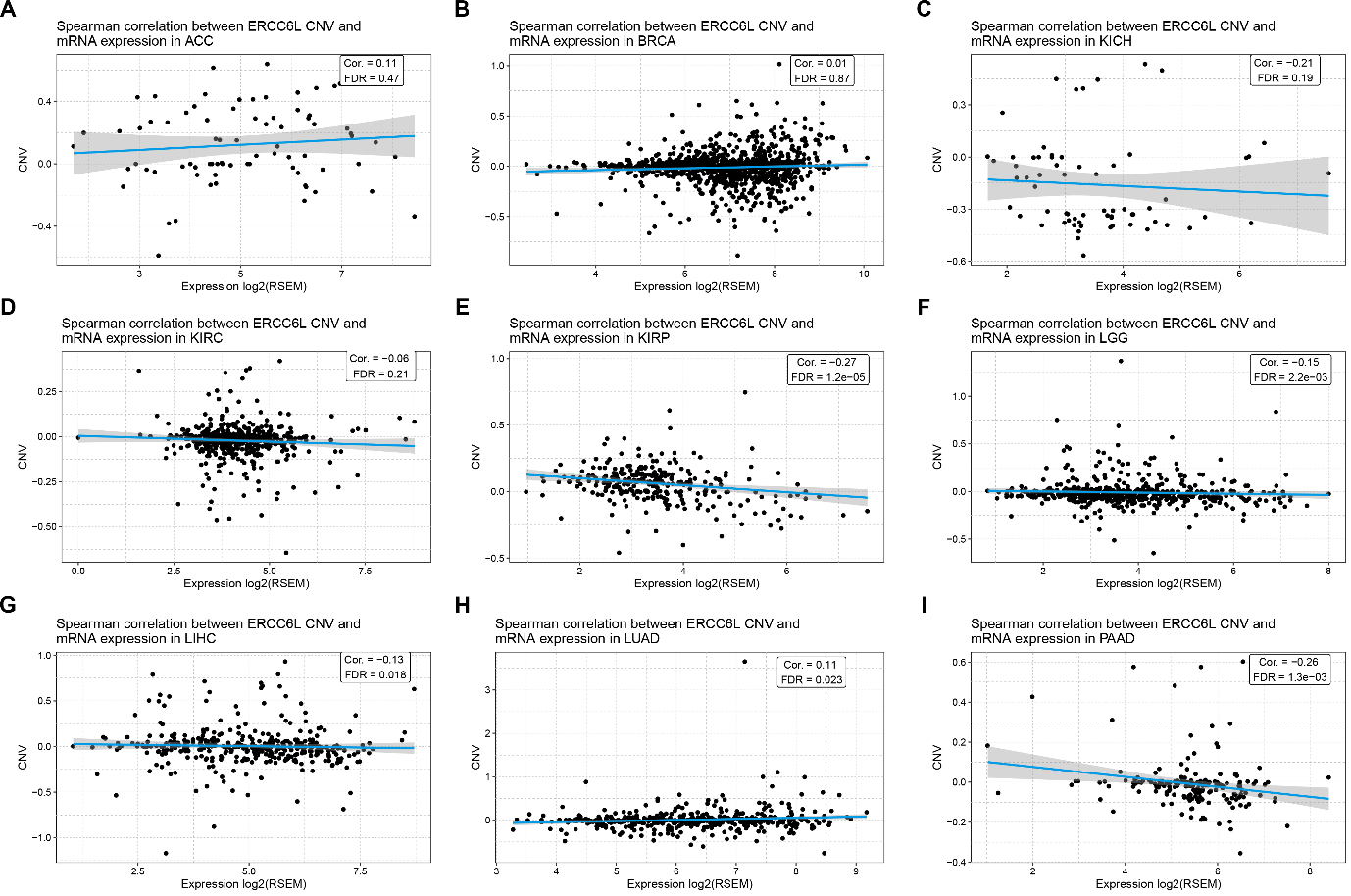
**

**Fig. S18 Correlation analysis of mRNA levels of ERCC6L and copy number variations in different cancers.**

(A-I) Plots indicating the correlations between copy number variation (CNV) and ERCC6L mRNA levels in patient samples from indicated cancer types.

**
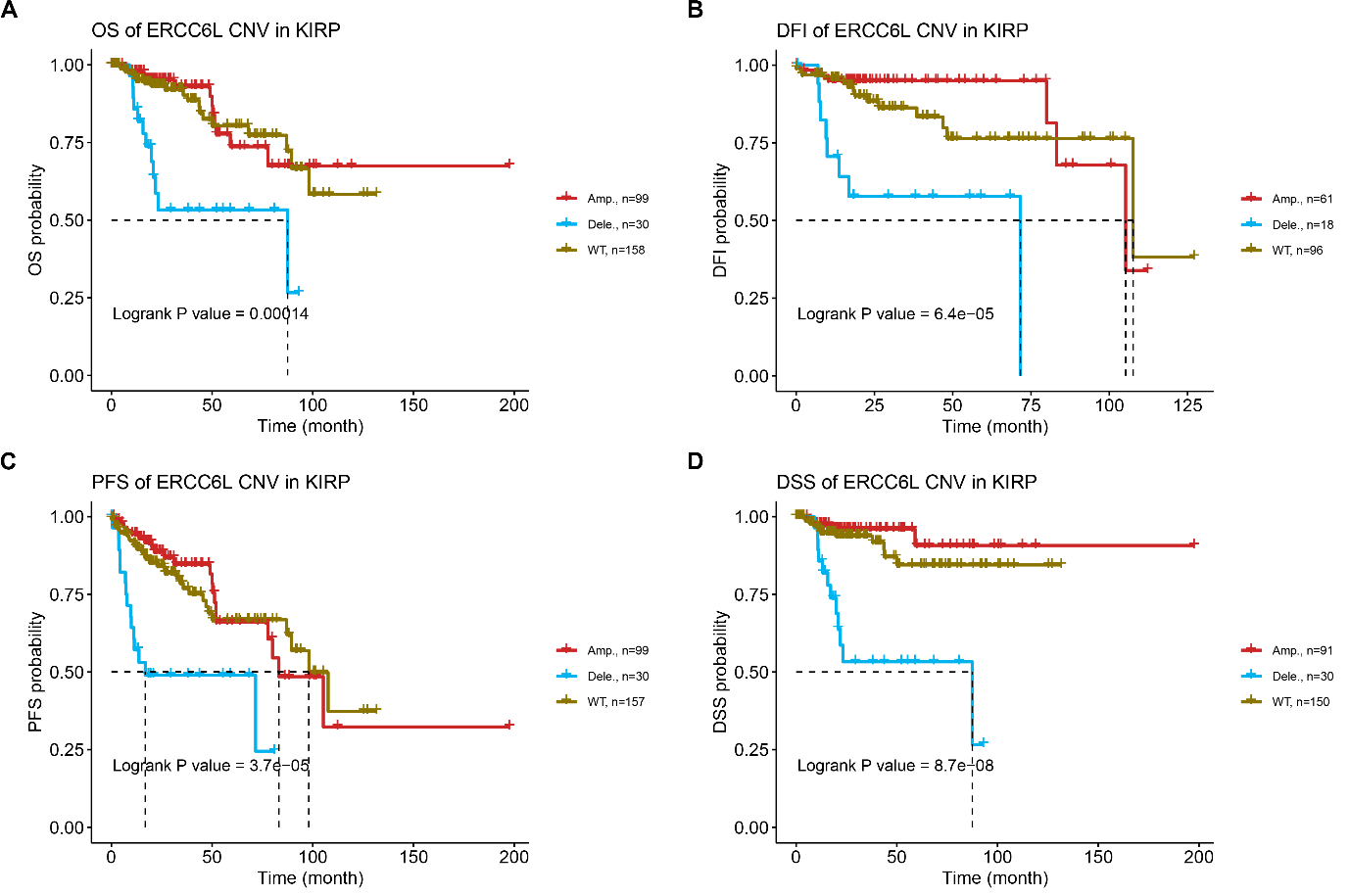
**

**Fig. S19 Patient survival analysis of ERCC6L CNV in KIRP.**

Survival probabilities (A, overall survival, OS; B, disease-free interval, DFI; C, progression-free survival; D, PFS and disease-specific survival, DSS) of KIRP patients subdivided by the CNV (amplification, AMP; deletion, Dele; wild type; WT) of ERCC6L gene.

**
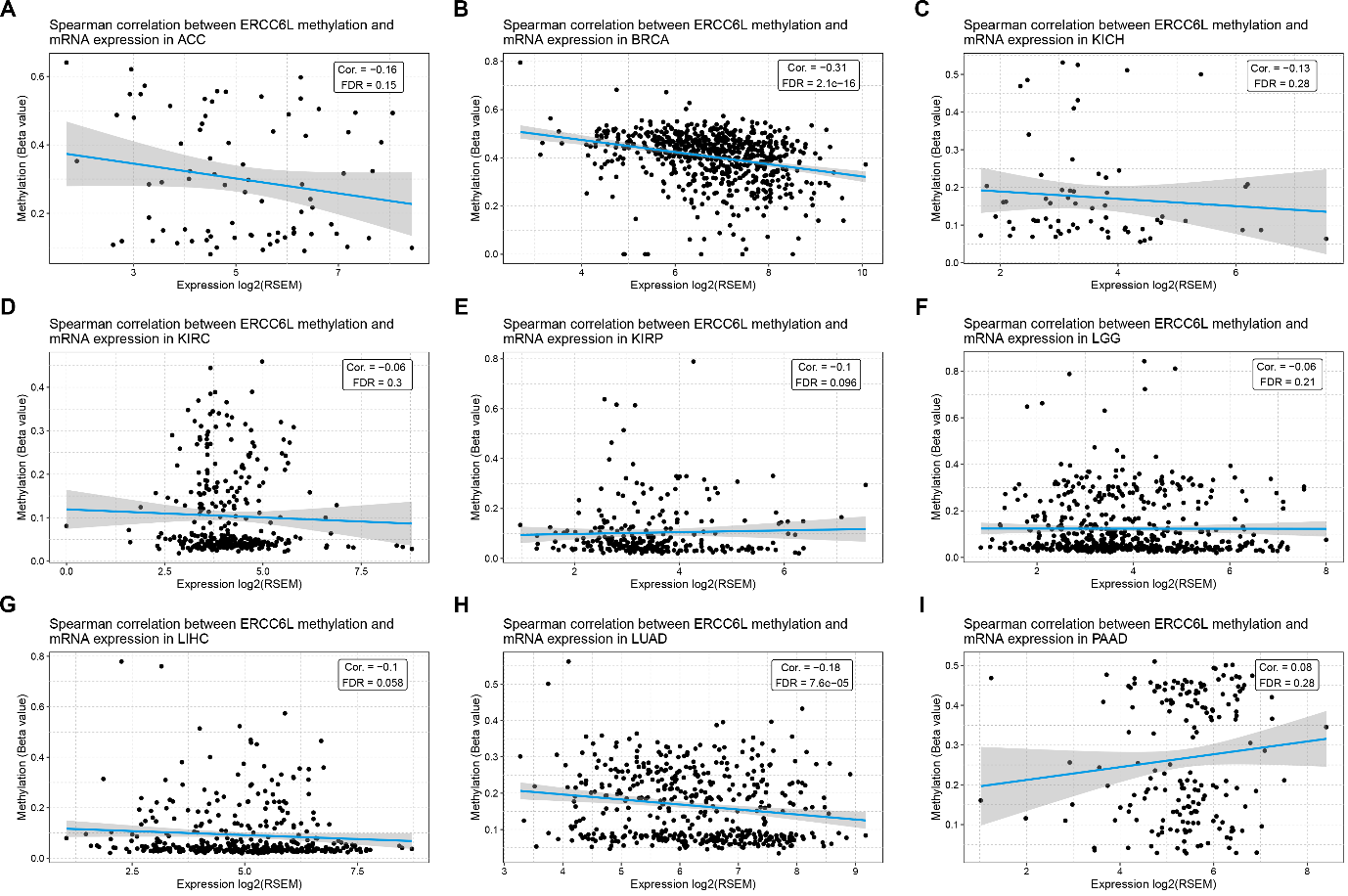
**

**Fig. S20 Correlation analysis of mRNA levels of ERCC6L and methylation of *ERCC6L* promoter in different cancer types.**

(A-I) Plots indicating the correlations between the methylation of ERCC6L promoter and ERCC6L mRNA levels in patient samples from indicated cancer types.

**
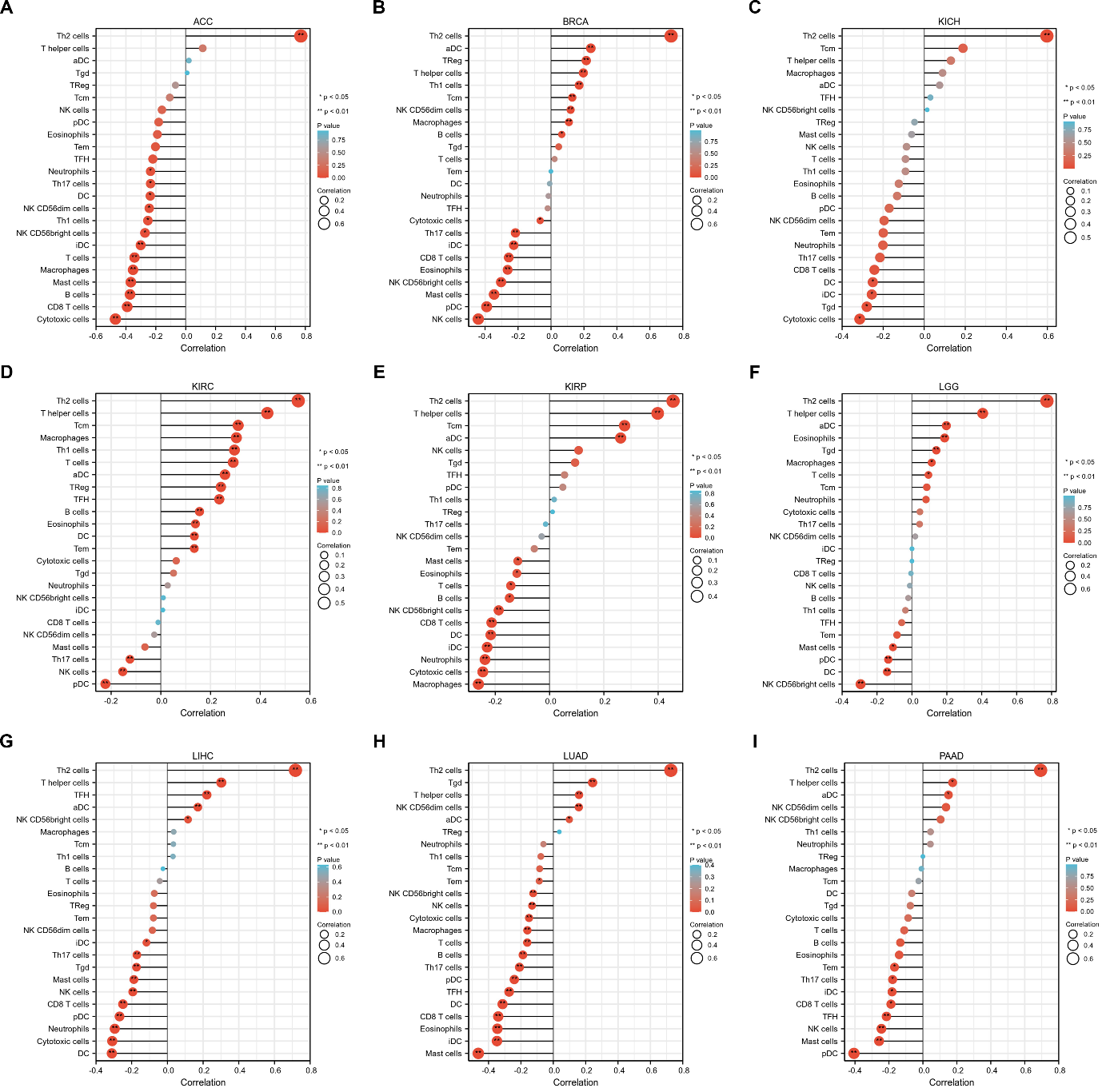
**

**Fig. S21 Correlations between ERCC6L expression and immune cell infiltration in different cancer types.**

(A-I) Plots showing the correlations between ERCC6L and the infiltration of indicated immune cells in patients with suggested cancers.

**
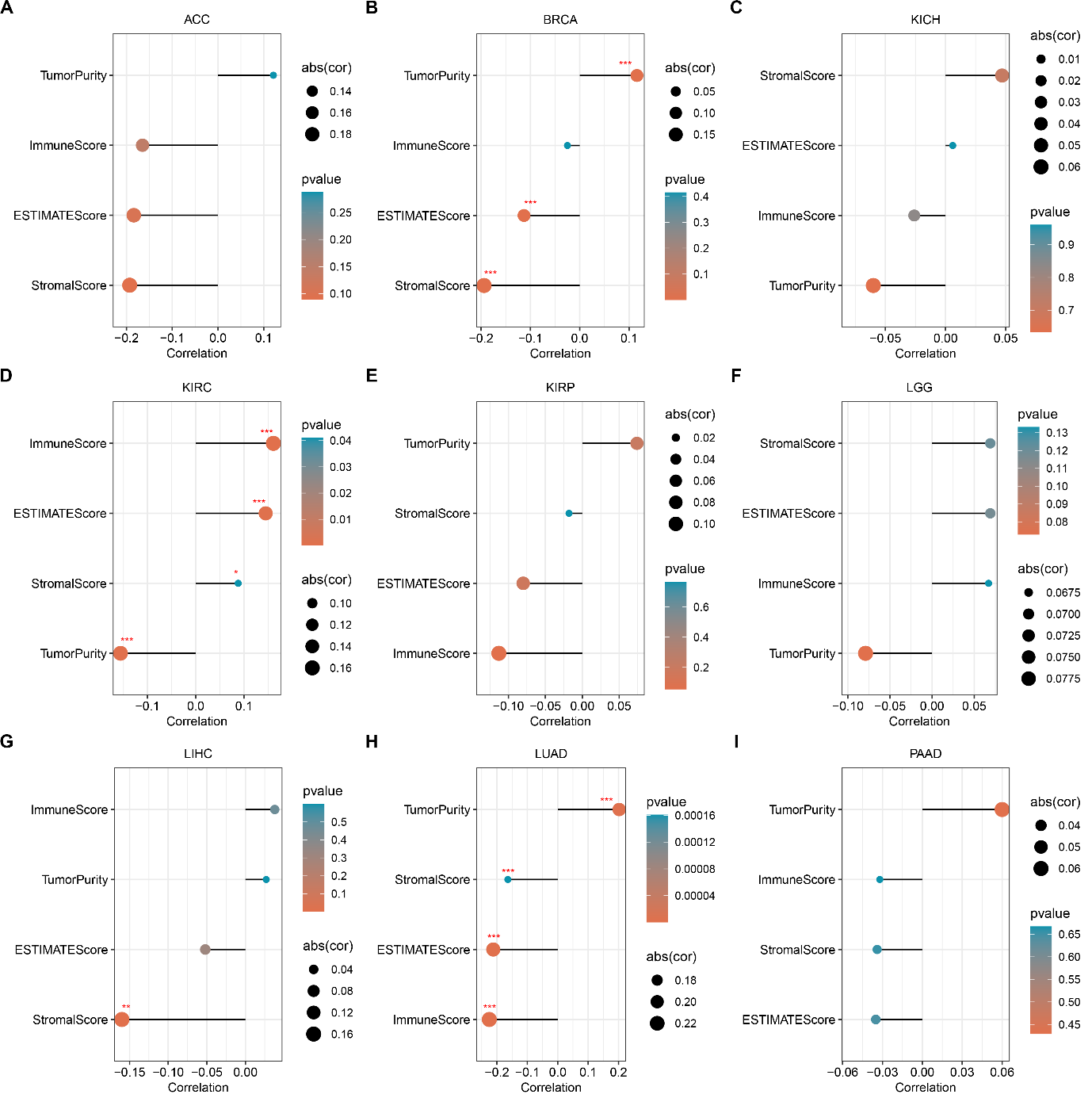
**

**Fig. S22 Correlations between ERCC6L expression and immune cell infiltration in different cancer types.**

(A-I) Plots showing the correlations between ERCC6L and the infiltration of indicated immune cells (as determined by ImmuneScore, TumorPuritym ESTIMATEScore and StromalScore) in patients with suggested cancers.


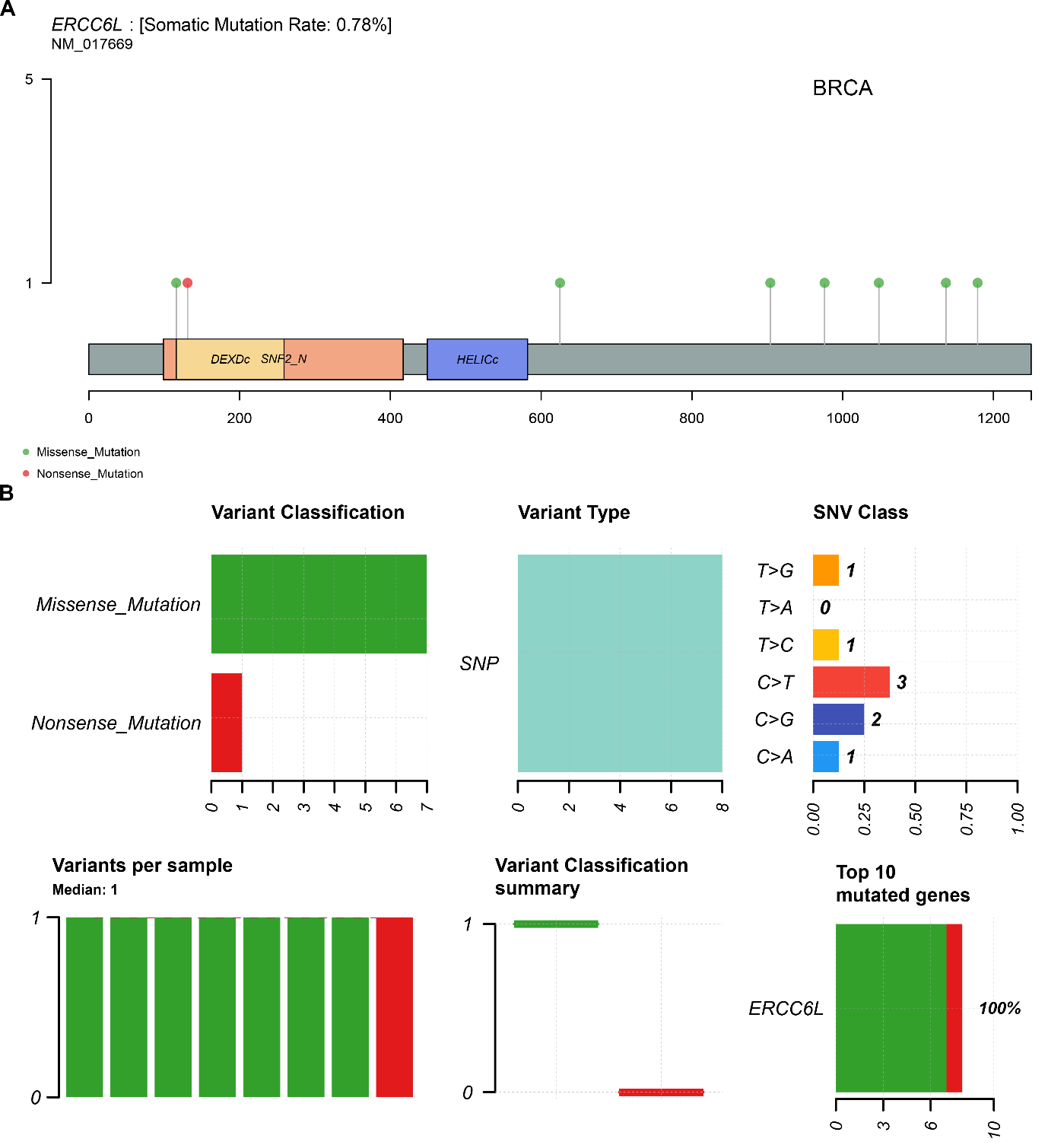


**Fig. S23 Analysis of somatic mutations of ERCC6L in BRCA.**

(A) Schematic plot showing the most common somatic mutation sites of ERCC6L gene in BRCA patients. (B) Classification and the percentage of mutation variants of ERCC6L gene in BRCA patients.


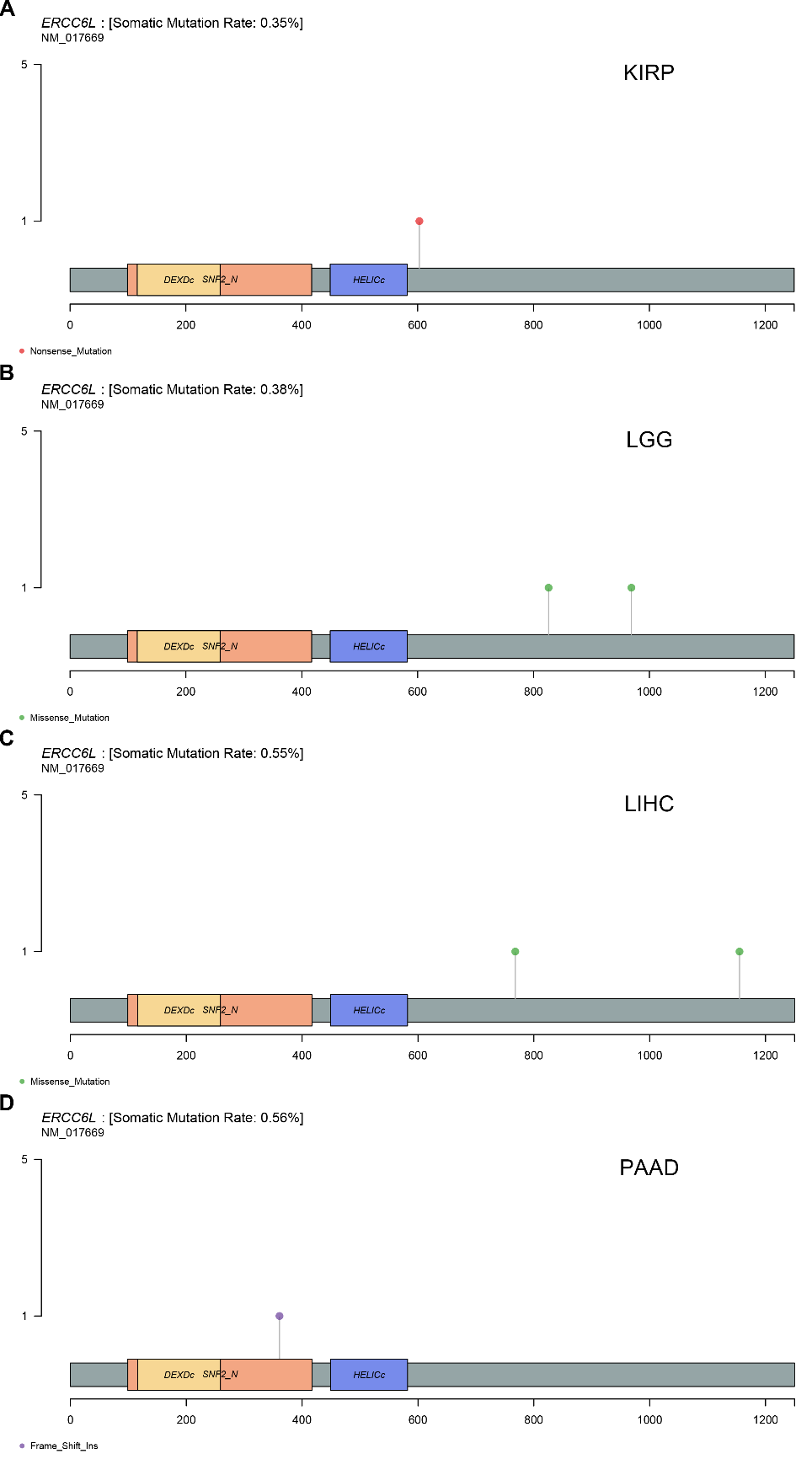


**Fig. S24 Analysis of somatic mutations of ERCC6L.**

Schematic plot showing the most common somatic mutation sites of ERCC6L gene in KIRP (A), LGG (B), LIHC (C) and PAAD (D).


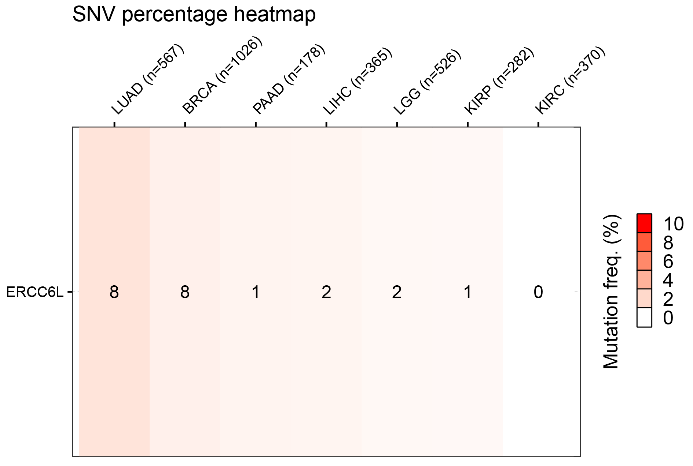


**Fig. S25 SNV frequencies of ERCC6L.**

Plot showing the SNV frequencies of ERCC6L in indicated cancers.


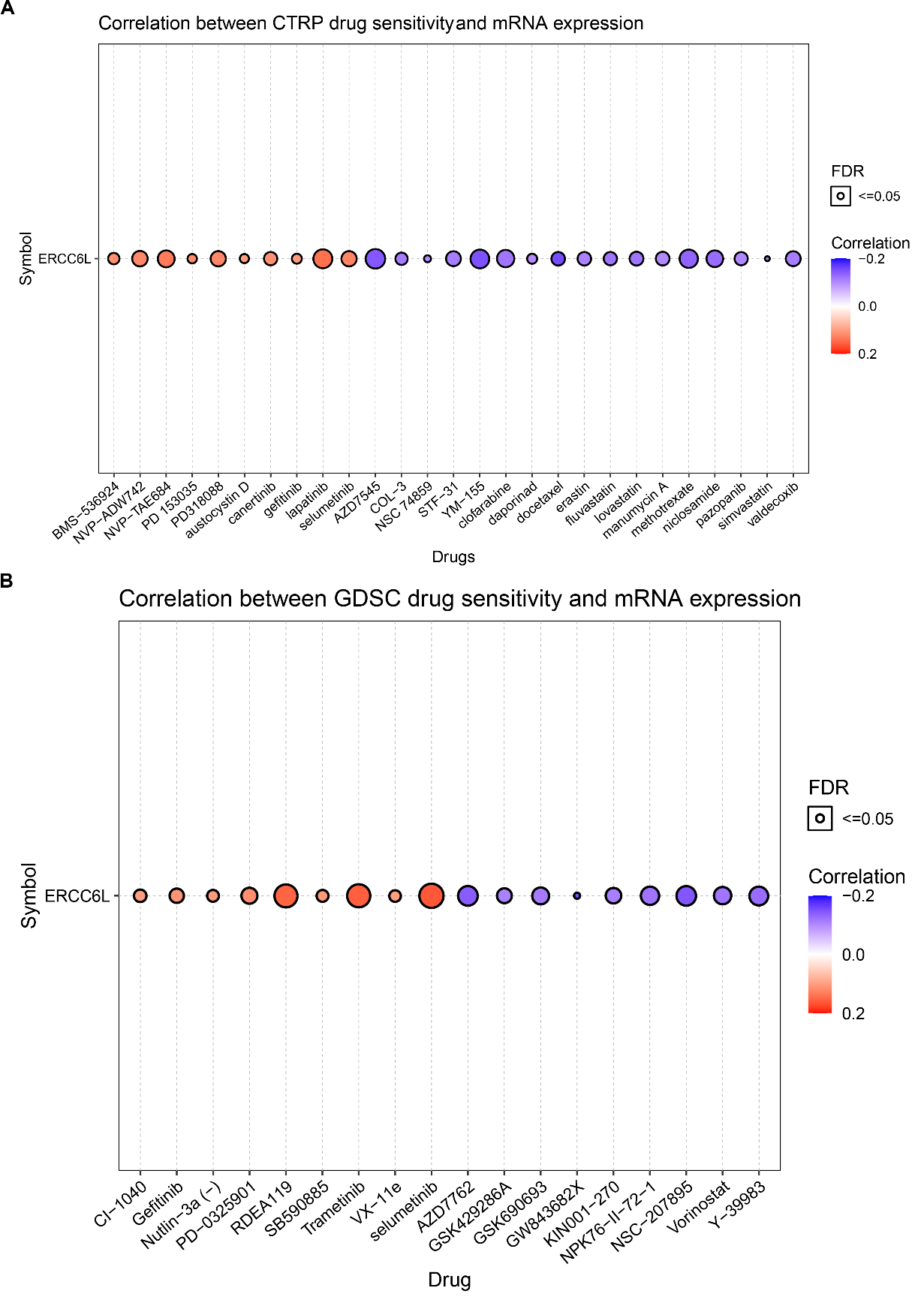


**Fig. S26. Correlations between ERCC6L expression and drug sensitivity.**

(A, B) Plots showing the associations between ERCC6L mRNA expression and the drug sensitivity from CTRP (A) and GDSC (B).
